# Tuning the rate of aggregation of hIAPP into amyloid using small-molecule modulators of assembly

**DOI:** 10.1038/s41467-022-28660-7

**Published:** 2022-02-24

**Authors:** Yong Xu, Roberto Maya-Martinez, Nicolas Guthertz, George R. Heath, Iain W. Manfield, Alexander L. Breeze, Frank Sobott, Richard Foster, Sheena E. Radford

**Affiliations:** 1 Astbury Centre for Structural Molecular Biology, School of Molecular and Cellular Biology, Faculty of Biological Sciences, University of Leeds, Leeds, LS2 9JT UK; 2 Astbury Centre for Structural Molecular Biology, School of Physics and Astronomy, University of Leeds, Leeds, LS2 9JT UK; 3 Astbury Centre for Structural Molecular Biology, School of Chemistry, University of Leeds, Leeds, LS2 9JT UK

**Keywords:** Biophysics, Biochemistry

## Abstract

Human islet amyloid polypeptide (hIAPP) self-assembles into amyloid fibrils which deposit in pancreatic islets of type 2 diabetes (T2D) patients. Here, we applied chemical kinetics to study the mechanism of amyloid assembly of wild-type hIAPP and its more amyloidogenic natural variant S20G. We show that the aggregation of both peptides involves primary nucleation, secondary nucleation and elongation. We also report the discovery of two structurally distinct small-molecule modulators of hIAPP assembly, one delaying the aggregation of wt hIAPP, but not S20G; while the other enhances the rate of aggregation of both variants at substoichiometric concentrations. Investigation into the inhibition mechanism(s) using chemical kinetics, native mass spectrometry, fluorescence titration, SPR and NMR revealed that the inhibitor retards primary nucleation, secondary nucleation and elongation, by binding peptide monomers. By contrast, the accelerator predominantly interacts with species formed in the lag phase. These compounds represent useful chemical tools to study hIAPP aggregation and may serve as promising starting-points for the development of therapeutics for T2D.

## Abstract (web summary)

Here the authors carry out chemical kinetic studies revealing that aggregation of hIAPP and its variant S20G involves secondary nucleation. Two small molecules with novel scaffolds are shown to inhibit or accelerate aggregation by binding different molecular species.

## Introduction

There are more than fifty protein-misfolding diseases characterised by the pathological self-assembly of peptides and proteins into amyloid fibrils, such as Alzheimer’s disease (AD), type 2 diabetes (T2D), Parkinson’s disease (PD) and systemic amyloidosis^1,2^. Human islet amyloid polypeptide (hIAPP or amylin) is a 37-residue neuropancreatic hormone that is co-secreted with insulin^3^. Its physiological functions include regulation of blood glucose homeostasis and prevention of gastric emptying through mediating the central nervous system^3,4^. Under pathophysiological conditions, hIAPP abnormally self-associates into amyloid fibrils and is the major protein constituent found in amyloid deposits in pancreatic islets in patients with T2D—a disease affecting more than 300 million individuals worldwide^3,5–7^. Pancreatic islet amyloidosis is believed to contribute to the progression of T2D^8,9^, β-cell loss^10^ and islet transplant failure^11^. The sequence of hIAPP is similar (*ca*. 50% similarity) to that of β-amyloid polypeptide (Aβ)^12^, and both hIAPP and Aβ have been detected in the brain plaques in AD^13^. Indeed, studies in vitro have shown that hIAPP can co-aggregate with Aβ^14–16^, and others have shown an epidemiological link between diabetes and neurodegenerative diseases including AD^13,17,18^, proposing an AD-associated type 3 diabetes (T3D)^19,20^. There is only one natural genetic mutation of hIAPP reported so far (the S20G variant)^21^. This variant is associated with early-onset T2D in the Japanese population and several in vitro experiments have demonstrated that S20G is more aggregation-prone compared with wild-type (wt) hIAPP^22–25^.

Despite being first identified as the amyloidogenic peptide associated with T2D more than 30 years ago^5,7^, and the recent solution of near-atomic resolution cryo-EM structures of wt hIAPP and S20G fibrils formed in vitro^26–28^, the aggregation mechanism of hIAPP is still unclear. Numerous studies of hIAPP fibrillation using synthetic peptide variants have illustrated the influence of particular residues or regions of the sequence on the aggregation processes (reviewed in^3^). Others have reported that the kinetics of hIAPP self-assembly follow a characteristic nucleation-dependent polymerisation process involving primary and secondary pathways, and highlighted the importance of secondary nucleation in fibril formation^29–32^. Recently, chemical kinetic analysis by global fitting of kinetic models has been used to determine the molecular mechanisms of AD-associated Aβ aggregation, including the effects of sequence^33–37^, pH^34^, agitation^38^ and chaperones^39^ on the relative contribution of the different processes in amyloid fibril formation (primary nucleation, secondary nucleation, elongation and fragmentation)^40^. By contrast, only a single report to date has applied this approach to study IAPP, using a non-natural variant in which the C-terminus lacks its natural amidation^32^: a moiety that forms key interactions in the fibril structures^26,27^ and is known to affect the rate of aggregation^41^ (note that a second report on the amidated variant was published while our manuscript was under consideration^42^). Even more intriguing is the S20G variant which, despite containing only a single amino acid substitution, forms different amyloid structures from wt hIAPP, including a novel three-protofilament structure which was postulated, based on its unusual asymmetric structure, to form via a secondary nucleation-dominated mechanism^26^.

Much effort has been directed towards the development of modulators (mostly inhibitors) against amyloid-forming proteins and peptides^43–45^, including hIAPP^46^. A plethora of inhibitors including peptide mimics^47–49^, small molecules^50,51^, inorganic metal ions^52^ and nanoparticles^53^ has been reported^46,54,55^. For example, a fibril structure of hIAPP has been used to design peptide inhibitors and these molecules were shown to inhibit fibril formation of hIAPP with low efficiency^28^. However, peptide mimics suffer from problems such as poor membrane permeability^56^ and proteolytic degradation^57^. Furthermore, many reported small molecules are based on flavanols or polyphenols which are known to exhibit poor stability (e.g. prone to oxidation^58,59^), poor selectivity^44^, poor pharmacokinetic properties (e.g. variable bioavailability^60^) or pan-assay interference (PAIN) properties^61^. Therefore, small molecules with more drug-like properties are needed. Given that the agents of toxicity in amyloid diseases (including T2D) are still unclear^62,63^, the discovery of small molecules that can either inhibit or enhance the aggregation rate of hIAPP offers important opportunities to delineate the mechanisms of fibril growth and amyloid-associated toxicity, as well as to act as starting-points of therapeutic strategies to combat disease.

Here we report the development of optimised ThT fluorescent bioassay conditions of wt hIAPP and S20G that enable a quantitative global chemical kinetic analysis of its assembly mechanism. We show using these assays that both peptides aggregate by a mechanism that is dominated by multi-step surface-catalysed secondary nucleation (over primary nucleation). Comparison of the different microscopic steps underlying aggregation of the two variants showed that more rapid primary and secondary pathways result in the enhanced aggregation rate of S20G. Via targeted screening of 1500 compounds, we also identify two small-molecule modulators of aggregation with chemical scaffolds distinct from previous modulators of hIAPP assembly^46,54,55^, one that delays amyloid formation (inhibitor YX-I-1), and a second which enhances the rate of aggregation (accelerator YX-A-1) of the peptide. Both molecules show high specificity: the inhibitor significantly delays the aggregation of wt hIAPP, but not S20G or Aβ42; while the accelerator enhances the aggregation of both IAPP peptides at substoichiometric concentrations, but has no effect on Aβ42. The kinetic analysis combined with native mass spectrometry, fluorescence spectroscopy, SPR and NMR analysis of the hIAPP:small-molecule interaction revealed that the inhibitor retards primary nucleation, secondary nucleation and elongation by binding to peptide monomers; whereas the accelerator likely interacts with species formed by primary nucleation in the lag phase. Overall, the results reveal two lead compounds able to modulate hIAPP aggregation which is promising agents for therapeutic intervention in T2D.

## Results

### Concentration-dependent aggregation kinetics of wt hIAPP and S20G

**Fig. 1:**
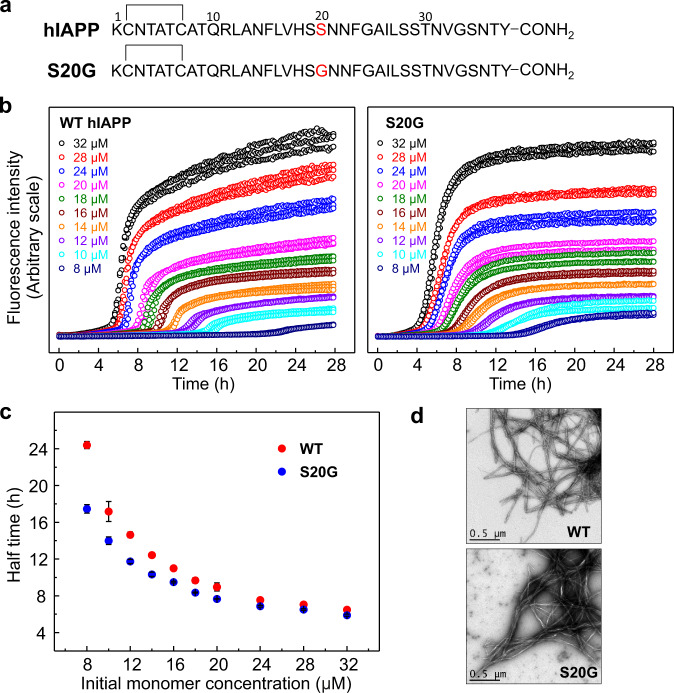
Concentration-dependent aggregation of wt hIAPP and S20G. **a** Sequences of wt hIAPP and the variant S20G. Both peptides have a disulfide bond between Cys-2 and Cys-7 and amidated C-termini. **b** Concentration-dependent aggregation kinetics of wt hIAPP and S20G. ThT fluorescence intensity of wt hIAPP and S20G is plotted over time at initial monomer concentrations ranging from 8 μM to 32 μM. Each experiment was performed three times with three technical replicates each time using different batches of the peptides. Samples contained different concentrations of peptide, 25 mM sodium phosphate buffer, 50 μM ThT and 2% (v/v) DMSO at pH 6.8 and were incubated at 30 °C quiescently. **c** Plots of half time (t_50_) versus the initial monomer concentration. Results are mean ± SD of *n* = 3. **d** TEM images of wt hIAPP and S20G fibrils taken from wells containing 10 μM peptide after the ThT fluorescence had reached a plateau (42 h). The scale bar is 0.5 μm. The TEM images are consistent in all three replicates.

The kinetics of amyloid formation of wt hIAPP and S20G, monitored by the amyloid-sensitive fluorescent probe, thioflavin T (ThT), are shown in Fig. 1. Each peptide was produced by microwave-assisted peptide synthesis and contained an amidated C-terminus so as to match the natural peptide sequence (Fig. 1a, Methods). The purity of the peptides was assessed by analytical high-performance liquid chromatography (HPLC) and the presence of the intramolecular disulfide bond between Cys-2 and Cys-7 was confirmed by mass spectrometry (Supplementary Fig. 1).

Before conducting a detailed kinetic analysis of hIAPP aggregation several key control experiments were performed. These included demonstration that (i) the presence of ThT does not affect the aggregation kinetics; (ii) the fluorescent signal is proportional to the mass concentration of amyloid fibrils formed;^33,34,40^ and (iii) the presence of DMSO that is required to solubilise the small molecules does not perturb the aggregation kinetics (Supplementary Fig. 2). Based on these results, a ThT concentration of 50 μM and DMSO concentration of 2% (v/v) were used in all assays. Other experimental conditions were also carefully controlled, including the preparation of degassed buffers, avoidance of introducing air bubbles, accurate control of the reaction temperature, and fibril growth under quiescent conditions.

The concentration-dependent aggregation of wt hIAPP and S20G were performed at initial monomer concentrations ranging from 8 μM to 32 μM. Highly reproducible curves were obtained for both peptides (Fig. 1b), resulting in sigmoidal fibril growth kinetics featuring a lag phase, growth phase and plateau phase, typical of nucleation-dependent fibril growth^29^ (Fig. 1b). The half time (t_50_) of aggregation, measured as the time when the ThT fluorescence intensity reached half of the fluorescence intensity between the baseline and the plateau, increases as the initial peptide concentration decreases (Fig. 1b,c). Compared with wt hIAPP, S20G aggregates more rapidly at all concentrations tested (Fig. 1c), consistent with previous reports^23–25^ that S20G has a higher aggregation propensity. Analysis of the product of aggregation by negative stain transmission electron microscopy (TEM) demonstrated amyloid-like fibrils with a long, straight morphology as the product of assembly (Fig. 1d).

### WT hIAPP and S20G aggregate predominantly through secondary nucleation

**Fig. 2:**
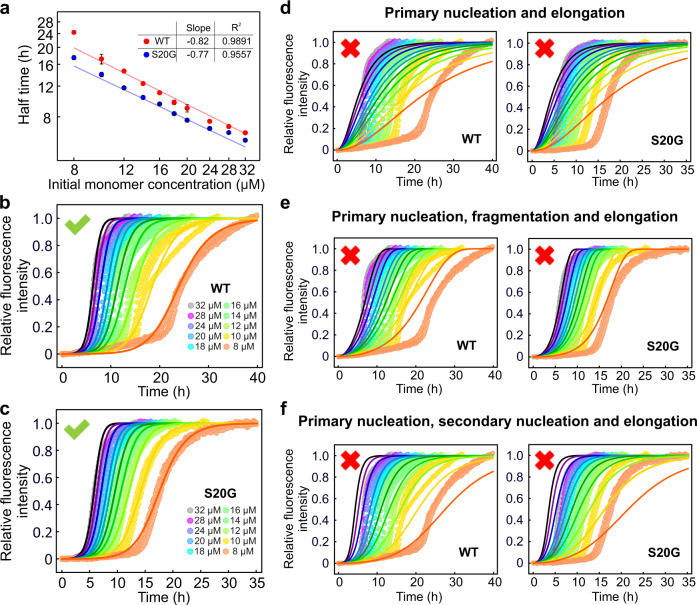
Global fitting of wt hIAPP and S20G aggregation kinetics to different kinetic models. **a** Double logarithmic plots of the half time of aggregation (t_50_) versus monomer concentration for wt hIAPP (red) and S20G (blue). The solid line is the fit of a power function (t_50_~Am_0_^γ^). The inset shows the average scaling exponent for wt hIAPP and S20G. Results are mean ± SD of *n* = 3. **b**, **c** Aggregation kinetics of wt hIAPP and S20G monitored by ThT fluorescence. Experimental data are shown as circles. The experiment at each concentration was performed in triplicate (shown in the same colour). The best global fit including primary nucleation, elongation and multi-step secondary nucleation is shown in lines. The model used for the global fitting includes three variable parameters: the combined rate constants *k*_+_*k*_n_ and *k*_+_*k*_2_ and Michaelis constant K_M_ for secondary nucleation. The reaction orders for primary (n_c_) and secondary nucleation (n_2_) were fixed to 2. Models containing primary nucleation and elongation (**d**); primary nucleation, elongation and fragmentation (**e**); and primary nucleation, elongation and secondary nucleation (**f**) each fail to achieve a satisfactory global fit for wt hIAPP and S20G.

We commenced quantitative analysis of the observed aggregation rate of wt hIAPP and S20G by determining the t_50_ and plotting this value as a function of the initial monomer concentration (m_0_). High dependence of the t_50_ on the initial monomer concentration is observed for both peptides which can be fitted by a power-law function (t_50_ ~ Am_0_^γ^) (Fig. 2a), as has been commonly observed in the aggregation kinetics of other aggregate-prone peptides^33,34,36^. In the power function, A is a constant and γ is defined as a scaling exponent that provides preliminary information about possible global fitting models^40^. As shown in Fig. 2a, the scaling exponents for wt hIAPP and S20G are similar (−0.82 and −0.77, respectively), suggesting that the aggregation mechanisms of the two peptides are not grossly different. At the highest concentrations assayed the experimental half times deviate from the power-law functions for both variants. This suggests that one or more saturating microscopic steps are involved at higher monomer concentrations, similar to previous observations for Aβ40^33^ and Aβ42^34^.

To gain insights into the mechanism of hIAPP fibril formation, we next performed global fitting of the ThT curves to dissect the rate constants of the individual microscopic steps using the online platform AmyloFit^40^. This platform applies the same rate constants and reaction orders to all the kinetic curves at all concentrations and generates combined rate constants, *k*_*+*_*k*_*n*_ and *k*_*+*_*k*_*2*_ (*k*_*+*_, *k*_*n*_ and *k*_*2*_ are the elongation rate constant, primary nucleation rate constant and secondary nucleation rate constant, respectively). Different kinetic models were fitted globally to the experimental data and various reaction orders for primary nucleation (n_c_) and secondary nucleation (n_2_) were also tried. The predication which best describes the experimental data includes primary nucleation, elongation and multi-step secondary nucleation (Fig. 2b, c; Supplementary Fig. 3). Models that do not include secondary nucleation failed to describe the experimental kinetic curves (Fig. 2d, e). Two models can be used to describe secondary nucleation: “secondary nucleation” and “multistep secondary nucleation”^33^. The latter treats the secondary nucleation as a multi-step process, in which peptide monomers first bind to the surface of the fibrils in a monomer concentration-dependent manner, followed by monomer concentration-independent steps, which could include conformational rearrangement, formation of a nucleus and detachment from the surface of the fibrils^33^. Without considering the multi-step effect of secondary nucleation, the model fails to reproduce the experimental data (Fig. 2f).

**Fig. 3:**
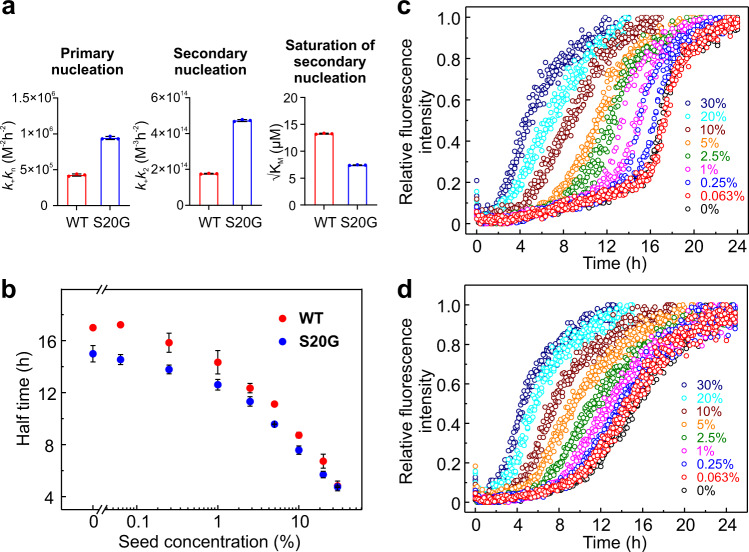
Parameters obtained by global fitting of wt hIAPP and S20G aggregation kinetics and seeding reactions. **a** The combined rate constants (*k*_+_*k*_n_ and *k*_+_*k*_2_) and monomer concentration of half-saturation of secondary nucleation (√K_M_) obtained from global fitting of the experimental kinetic data shown in Fig. 2 for wt hIAPP (red) and S20G (blue). Bars represent the mean and error bars show standard deviation (*n* = 3), with values for each replicate shown as points. **b** Half time (t_50_) as a function of the logarithm of the seed concentration for wt hIAPP (red) and S20G (blue). Results are mean ± SD of *n* = 3. Aggregation kinetics of **c** wt hIAPP and **d** S20G in the presence of preformed self-seeds. 10 μM monomer wt hIAPP/S20G was incubated with 0%, 0.063%, 0.25%, 1%, 2.5%, 5%, 10%, 20% or 30% (v/v) preformed self-seeds in 25 mM sodium phosphate buffer, pH 6.8 at 30 °C quiescently. Each experiment was performed twice with three technical replicates each time using different batches of the peptides.

The model that best describes the kinetic curves generates the combined rate constants (*k*_*+*_*k*_*n*_ and *k*_*+*_*k*_*2*_). This analysis showed that *k*_*+*_*k*_*n*_ and *k*_*+*_*k*_*2*_ of S20G are ~ 2-fold larger than those of wt hIAPP, consistent with its more rapid assembly into amyloid (Fig. 3a). For both variants the combined rate constant for secondary nucleation, *k*_*+*_*k*_*2*_, is larger than the combined rate constant *k*_*+*_*k*_*n*_ containing primary nucleation, such that *k*_*2*_/*k*_*n*_ > 10^8^. This indicates that most of the new aggregates for both variants are formed through surface-catalysed secondary nucleation rather than through primary nucleation. Such a scenario was previously observed for different Aβ peptide sequences^33,34,36^. Fitting the data to a multi-step secondary nucleation model also yields the parameter √K_M_ which reports on the monomer concentration of half-saturation of secondary nucleation^33^. The √K_M_ is lower for S20G than wt hIAPP (Fig. 3a), indicating a higher binding affinity of the S20G monomer to its fibril surface compared with wt hIAPP, consistent with the increased rate of secondary nucleation for this variant.

To verify the global fitting model and confirm the role of secondary nucleation in wt hIAPP/S20G assembly, we also analysed the kinetics of seeded fibril growth for each peptide by adding fibril seeds (0–30% (v/v)) of each peptide at the beginning of the aggregation reaction. It is reported that the fibril elongation process is negligible at a lower concentration of fibril seeds, whereas the lag time can be significantly shortened if there are secondary processes to catalyse the generation of new seeds^34,40^. As expected, the aggregation rate of wt hIAPP and S20G was enhanced significantly even in the presence of low concentrations of fibril seeds (Fig. 3b–d), in accord with the importance of secondary nucleation (over primary nucleation) in the aggregation kinetics of both peptides. To further validate the importance of elongation in fibril growth, we compared the aggregation kinetics of wt hIAPP and S20G in the presence of long (without sonication) or short (with sonication) fibril seeds (Supplementary Fig. 4a, d). The results showed that the t_50_ is decreased and the apparent growth rate increased for both wt hIAPP and S20G when the seeds are fragmented (more fibril ends) (Supplementary Fig. 4b, e and Supplementary Fig. 4c, f, respectively), consistent with elongation processes contributing to aggregation of both proteins.

### Identification of small-molecule modulators of hIAPP assembly

**Fig. 4:**
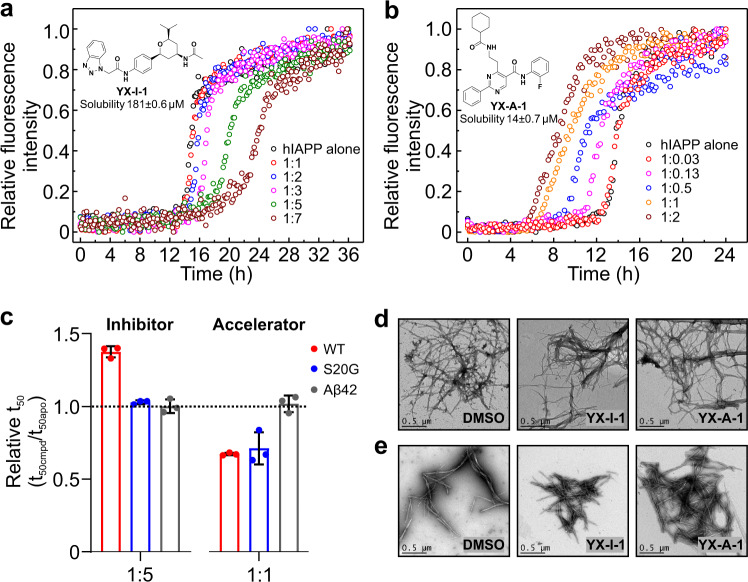
Small-molecule modulators which alter the aggregation kinetics of hIAPP. **a** Dose-dependent inhibition of wt hIAPP aggregation by YX-I-1. Relative ThT fluorescence intensity of 10 μM wt hIAPP in the absence (black) or presence of a 1:1 (red), 1:2 (blue), 1:3 (pink), 1:5 (green) or 1:7 (wine) molar ratio of wt hIAPP to YX-I-1 (25 mM sodium phosphate, pH 6.8, 30 °C, quiescently). Inset shows the chemical structure of YX-I-1 and its solubility. **b** Dose-dependent acceleration of wt hIAPP aggregation by YX-A-1. Relative ThT fluorescence intensity of 10 μM wt hIAPP in the absence (black) or presence of a 1:0.03 (red), 1:0.13 (pink), 1:0.5 (blue), 1:1 (yellow) or 1:2 (wine) molar ratio of wt hIAPP to YX-A-1 (25 mM sodium phosphate, pH 6.8, 30 °C, quiescently). Inset is the chemical structure of YX-A-1 and its solubility. **c** Relative t_50_ of wt hIAPP (red), S20G (blue) or Aβ42 (grey) aggregation in the absence or presence of YX-I-1 or YX-A-1. YX-I-1 delays the formation of wt hIAPP fibrils, but not S20G; while YX-A-1 enhances the fibril formation of both IAPP sequences. The compounds have no effect on the aggregation of Aβ42 (calculated from Supplementary Fig. 10c,d). *X*-axis indicates the molar ratio of the corresponding peptide to YX-I-1 (inhibitor) or YX-A-1 (accelerator). Relative t_50_ = t_50_(compound)/t_50_(DMSO). Bars represent the mean and error bars show standard deviation (*n* = 3), with values for each replicate shown as points. **d** TEM images of 10 μM wt hIAPP incubated with DMSO, 50 μM YX-I-1 or 20 μM YX-A-1. Samples were taken from 96-well plates after ThT fluorescence reached a plateau. Scale bar represents 0.5 μm. **e** TEM images of 10 μM S20G incubated with DMSO, 50 μM YX-I-1 or 10 μM YX-A-1. Samples were taken from 96-well plates after ThT fluorescence reached plateau. Scale bar represents 0.5 μm. Does-dependent experiments were performed twice with three technical replicates each time using different batches of the peptides. The effect of the inhibitor (hIAPP:YX-I-1 = 1:5) and accelerator (hIAPP:YX-A-1 = 1:1) on hIAPP aggregation at single concentration was determined more than three biological replicates independently. The TEM images are consistent in all three replicates.

To search for small molecules able to modulate the amyloid formation of wt hIAPP, we carried out targeted screening of 1500 compounds from the protein–protein interaction (PPI) library using a combinatorial approach of native electrospray ionisation mass spectrometry (nESI-MS) and ThT kinetic assays (see Methods and Supplementary Fig. 5) (a full description of the library and screening results will be presented elsewhere). The library was generated by PPI-NET based on an extensive analysis of chemical agents known to inhibit protein–protein interactions (PPIs) and includes novel chemotypes that are featured with PPI-privileged building blocks and fragments. Two molecules (YX-I-1 and YX-A-1) from the screening were selected for detailed analysis based on satisfying two criteria: (i) the ability to bind to monomers/oligomers of wt hIAPP using nESI-MS^64^ and (ii) significant alteration of the t_50_ of aggregation kinetics measured using ThT fluorescence. Importantly, YX-I-1 and YX-A-1 are structurally distinct from previously reported modulators of hIAPP assembly^46,54,55^, and have more drug-like properties that satisfy Lipinski’s rules (Supplementary Table 1). Dose-dependent analyses of the aggregation rates showed that YX-I-1 inhibits the aggregation of wt hIAPP, extending the t_50_ from 15.1 ± 0.2 h to 23.4 ± 1.0 h at a 1:7 molar ratio of peptide:inhibitor (Fig. 4a). By contrast, YX-A-1 accelerates wt hIAPP assembly, even when added in substoichiometric concentration ratios (e.g. at a peptide:YX-A-1 molar ratio of 1:0.5 the t_50_ is reduced ~1.5-fold) (Fig. 4b). Increasing the concentration of YX-A-1 further accelerates aggregation, until saturation is achieved at an *ca*. 1:1 molar ratio of peptide:YX-A-1 (Fig. 4b). At the end of each reaction, fibrils were observed using negative stain TEM (Fig. 4d). We further analysed the height and length distributions of fibrils formed in the presence of DMSO, YX-I-1 or YX-A-1 by AFM (Supplementary Fig. 6a), which can provide a quantitative comparison of fibril morphology. Fibrils formed in the presence of YX-A-1 have lower fibril height (*p* < 0.0001) than those formed in the presence of DMSO; while fibrils formed in the presence of YX-I-1 have similar fibril height as the DMSO-treated samples (*p* = 0.2564) (Supplementary Fig. 6b). When comparing the fibril length, those formed in the presence of YX-I-1 (*p* = 0.3153) or YX-A-1 (*p* = 0.1190) have similar fibril length as those formed in the presence of DMSO (Supplementary Fig. 6c). These data suggest that YX-A-1 may change the course of aggregation and result in different fibril structures, although we note that full structure determination using cryo-EM will be needed to confirm this hypothesis.

To ensure that the small molecules themselves do not self-assemble in buffer, and hence to show that the activity of the small molecules is not attributable to their self-assembly, as has been observed for several other small-molecule modulators of amyloid assembly^64,65^, the solubility of the compounds in the buffer used was determined, along with their possible association using dynamic light scattering (DLS) and NMR. The solubility of YX-I-1 and YX-A-1 under the experimental conditions employed were found to be 181 ± 0.6 μM and 14 ± 0.7 μM, respectively. The scattering intensity of solutions of YX-I-1 or YX-A-1 was also similar to that of a DMSO control, demonstrating that the two small molecules do not form particles^66^ (Supplementary Fig. 7a). To further assess the self-assembly propensity of the two molecules, an NMR approach based on quantifying the 1D-^1^H intensity of a well-separated ^1^H resonance at various concentrations was exploited^67^. For both molecules, the intensity increased linearly with concentration, indicating micelle formation did not occur (Supplementary Fig. 7b). The inhibitory potential of small molecules against amyloid aggregation can be highly dependent on the experimental conditions. For example, the widely reported anti-amyloid pan-inhibitor EGCG is ineffective as an inhibitor of α-synuclein aggregation when the pH is decreased from pH 7 to pH 6^68^. The inhibitory potential of YX-I-1 against wt hIAPP aggregation was thus tested at lower (pH 6.4) and higher (pH 7.4) pH values. These experiments showed that YX-I-1 inhibits wt hIAPP aggregation at all pH values tested, even though the aggregation rate of wt hIAPP is pH dependent^69^ (Supplementary Fig. 8a, b). Similarly, the acceleration effect of YX-A-1 was also maintained at both pH values (Supplementary Fig. 8c, d). The end products of the reaction were assessed using TEM, and showed the formation of amyloid fibrils under all conditions explored (Supplementary Fig. 8e, f). Further experiments (see Methods) showed that the two small molecules do not bind to wt hIAPP fibrils and did not result in fibril depolymerisation (at least over 40 h) (Supplementary Fig. 9).

To determine whether the small molecules are also able to modulate the aggregation of S20G, and hence to determine how the aggregation mechanisms of the two peptides vary at a molecular level, the effect of YX-I-1 and YX-A-1 on the time course of S20G aggregation was also monitored using ThT fluorescence. The results were striking, showing that YX-I-1 had no effect on the t_50_ of S20G aggregation, despite this peptide differing from wt hIAPP in only a single residue (Fig. 4c, also Supplementary Fig. 10a). This indicates a highly specific interaction of the small molecule with one or more species formed during the self-assembly process of wt hIAPP and highlights the importance of residue Ser-20 in defining the course of assembly. By contrast, YX-A-1 was found to accelerate the aggregation of both wt hIAPP and S20G (Fig. 4c, also Supplementary Fig. 10b). Again, TEM showed the formation of amyloid fibrils in reactions of S20G treated with each compound (Fig. 4e).

As a final test of the specificity of the two modulators, we also evaluated their effect on the aggregation of Aβ42, a stringent control of specificity since wt hIAPP and Aβ42 have related sequences^12^ (25% identity/50% similarity) and similar fibril structures^26,27^. We performed the Aβ42 assays under two conditions: (1) conditions which have previously been reported to generate highly reproducible aggregation kinetics of Aβ42 and have been used widely to determine the effect of small molecules on Aβ42 aggregation^70^; (2) identical conditions to those used here to monitor the aggregation of hIAPP (see Methods). Neither molecule significantly altered the kinetics of Aβ42 aggregation under these conditions, highlighting the high specificity of the two compounds for hIAPP (Fig. 4c and Supplementary Fig. 10c–e).

### Quantitative kinetic analysis of wt hIAPP aggregation in the presence of inhibitor YX-I-1

**Fig. 5:**
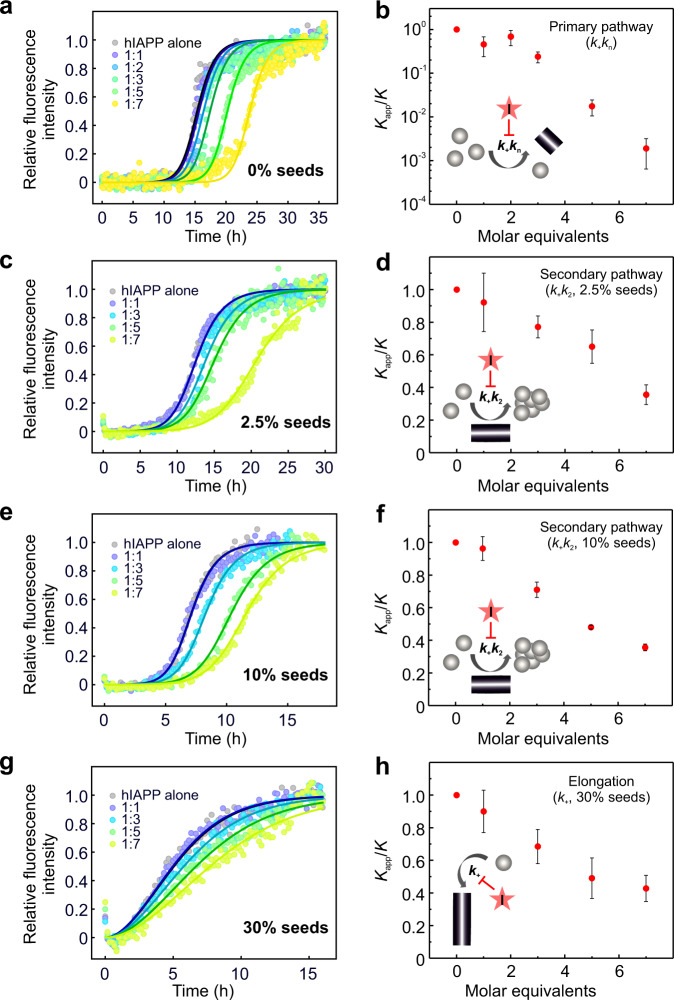
YX-I-1 inhibits primary nucleation, secondary nucleation and elongation of wt hIAPP. Aggregation kinetics of 10 μM wt hIAPP in the absence or presence of a 1:1, 1:2, 1:3, 1:5 or 1:7 molar ratio of wt hIAPP:YX-I-1 are shown in the presence of different amounts of preformed fibril seeds. In each plot the solid lines show predications from global fitting of the experimental data as indicated. **a** Aggregation kinetics in the absence of fibril seeds with different molar ratios of hIAPP:YX-I-1, as indicated. The solid lines show fits in which the combined rate constant for primary nucleation and elongation (*k*_+_*k*_n_) was varied. **b** Changes in *k*_+_*k*_n_ in the presence of increasing concentrations of YX-I-1 relative to the rate constants of aggregation reaction treated with DMSO alone. *K* is the combined rate constant *k*_+_*k*_n_ in the presence of DMSO; *K*_app_ is the combined rate constant *k*_+_*k*_n_ in the presence of YX-I-1. Aggregation kinetics in the presence of 2.5% (v/v) (**c**) or 10% (v/v) (**e**) preformed fibril seeds with different molar ratios of hIAPP:YX-I-1, as indicated. The solid lines show fits in which the combined rate constant for secondary nucleation and elongation (*k*_+_*k*_2_) was varied. **d**, **f** Changes in *k*_+_*k*_2_ in the presence of increasing concentrations of YX-I-1 relative to the rate constants of aggregation reaction treated with DMSO alone. *K* is the combined rate constant *k*_+_*k*_2_ in the presence of DMSO; *K*_app_ is the combined rate constant *k*_+_*k*_2_ in the presence of YX-I-1. **g** Aggregation kinetics in the presence of 30% (v/v) preformed fibril seeds with different molar ratios of hIAPP:YX-I-1, as indicated. The solid lines show fits in which only the elongation rate constant (*k*_+_) was varied. **h** Changes in *k*_+_ in the presence of increasing concentrations of YX-I-1 relative to the rate constant of aggregation reaction treated with DMSO alone. *K* is the elongation rate constant *k*_+_ in the presence of DMSO; *K*_app_ is the elongation rate constant *k*_+_ in the presence of YX-I-1. All the experiments were performed in 25 mM sodium phosphate, pH 6.8 at 30 °C, quiescently. Results are mean ± SD of *n* = 3.

Having established that YX-I-1 can inhibit the aggregation of wt hIAPP in a dose-dependent manner, we next sought to determine the inhibited microscopic steps underlying the macroscopic aggregation events. Fitting the experimental ThT fibril growth curves in the presence of different concentrations of YX-I-1 into different chemical kinetic models^70,71^ revealed that a regime in which the combined rate constant including primary nucleation and elongation (*k*_*+*_*k*_*n*_) is specifically reduced by YX-I-1 reproduced the experimental data, indicating that YX-I-1 predominantly modulates the primary nucleation and elongation pathway (*k*_*+*_*k*_*n*_) (Fig. 5a). Indeed, the combined rate constant (*k*_*+*_*k*_*n*_) is decreased *ca*. 1000-fold at a peptide:inhibitor molar ratio of 1:7, compared with treatment with DMSO alone (Fig. 5b).

To investigate the possible effect of the inhibitor on secondary nucleation (*k*_*2*_) and elongation (*k*_*+*_), a series of aggregation kinetic experiments was carried out in the presence of different amounts of preformed fibril seeds and various concentrations of YX-I-1. When a small number of seeds (2.5% or 10% (v/v)) were introduced into the reaction mixtures, sigmoidal kinetic curves were obtained with greatly shortened t_50_, as expected for a reaction dominated by secondary nucleation and elongation (Fig. 5c, e, also Supplementary Fig. 11b, c). The addition of YX-I-1 led to concentration-dependent retardation of wt hIAPP aggregation in these seeded reactions, with an extended t_50_ (Fig. 5c, e, also Supplementary Fig. 11b, c). Quantitative analysis by globally fitting the experimental data yielded an excellent fit when the combined rate constant of secondary nucleation/elongation (*k*_+_*k*_2_) is reduced with increasing concentrations of the inhibitor, with the rate constant (*k*_+_*k*_2_) decreasing by approximately 60% at a 1:7 molar equivalent of wt hIAPP and YX-I-1 (Fig. 5d, f).

When 30% (v/v) preformed wt hIAPP seeds were added to 10 μM wt hIAPP monomers, the primary and secondary nucleation processes were bypassed and the elongation of the fibril seeds was the dominant mechanism as evident from the immediate increase of the ThT fluorescence. (Fig. 5g, Supplementary Fig. 11d). Under these conditions the addition of the inhibitor had a small, but significant effect on the t_50_ (Fig. 5g, Supplementary Fig. 11d), similar to the effect of a reported small-molecule inhibitor on Aβ42 elongation^72^. Fitting these data to a model in which only the elongation rate constant is affected by the inhibitor showed a decrease in the elongation rate constant by about 50% in the presence of a 1:7 molar equivalent of hIAPP:inhibitor (Fig. 5h). Together, the results provide clear kinetic evidence that YX-I-1 retards primary nucleation pathways (*k*_+_*k*_n_), secondary pathways (*k*_+_*k*_2_) and fibril elongation (*k*_+_). Considering the species involved in these different microscopic events^71^, the results suggest that the inhibition of wt hIAPP aggregation by YX-I-1 results from the interaction of the small molecule with wt hIAPP monomers.

### Quantitative kinetic analysis of wt hIAPP aggregation in the presence of YX-A-1

To identify the microscopic steps by which YX-A-1 accelerates wt hIAPP aggregation, the seeded growth experiments were repeated in the presence of increasing concentrations of the accelerator. The results of these experiments contrast markedly with those obtained with YX-I-1, with the acceleration of aggregation by YX-A-1 no longer occurring in the presence of seeds (Supplementary Fig. 12). Since secondary pathways dominate the seeded reactions at low seed concentrations (2.5% and 10% (v/v) seed) and elongation at the higher concentration of seed used (30% (v/v)), the results suggest that YX-A-1 most likely targets oligomeric hIAPP species formed during the lag phase, where primary nucleation processes are dominant. Such a finding is consistent with the substoichiometric activity of YX-A-1. Notably, the different effects observed in the unseeded and seeded experiments suggest that oligomeric species generated from primary nucleation and secondary nucleation have different structural features. Since YX-A-1 accelerates both wt hIAPP and S20G, however, the early oligomeric species of the two peptide sequences presumably must be similar.

### The molecular interactions of YX-I-1 and YX-A-1 with wt hIAPP

**Fig. 6:**
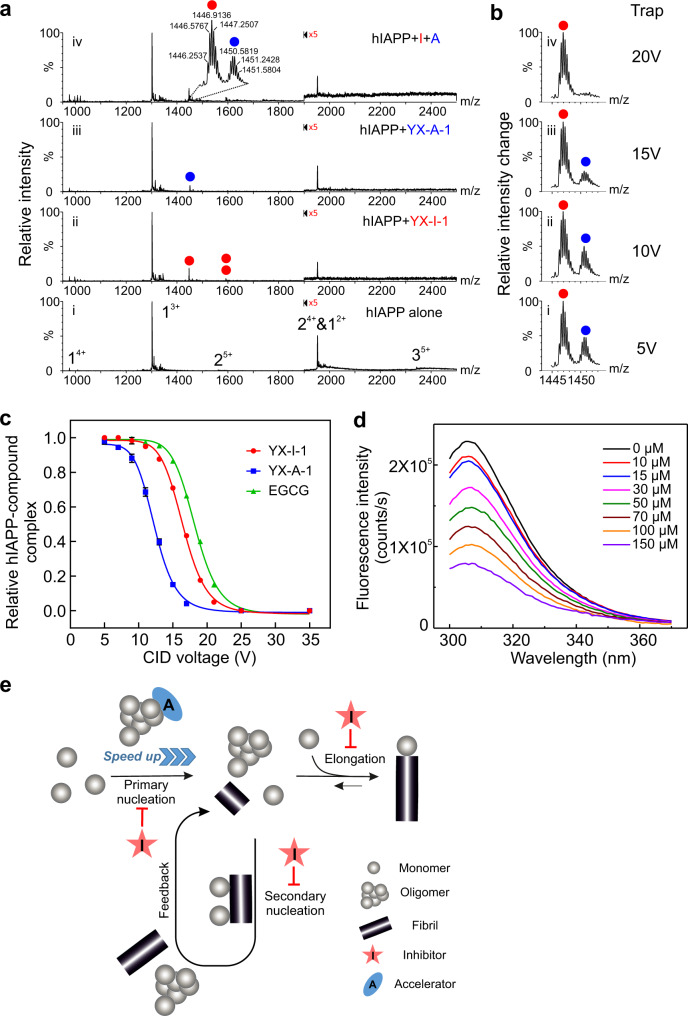
The interaction between wt hIAPP monomers and the two small-molecule modulators characterised by nESI-MS. **a** Positive-ion nESI-mass spectra of 16 μM hIAPP in the presence of (i) 2% (v/v) DMSO alone; (ii) with added YX-I-1 (molar ratio of wt hIAPP:YX-I-1 = 1:5); (iii) YX-A-1 (molar ratio of wt hIAPP:YX-A-1 = 1:2) or (iv) in an equimolar mixture of YX-I-1 and YX-A-1 (molar ratio of hIAPP:YX-I-1:YX-A-1 = 1:2.5:2.5). Bound peaks are denoted with circles (inhibitor YX-I-1 in red and accelerator YX-A-1 in blue) and the number of circles represents the number of small molecules bound. **b** Spectra obtained of the wt hIAPP-ligand^3+^ complex obtained in Fig. 6a (iv) at different trap collision energy to sample relative gas-phase stabilities of ligand binding. **c** Collision-induced dissociation (CID) of hIAPP-compound^3+^ complex. Intensity of hIAPP-compound^3+^ complex (hIAPP-compound^3+^/total hIAPP ion) plotted against the trap voltage. EGCG was included as a control as it is a known inhibitor of hIAPP aggregation^73^. 16 μM wt hIAPP was incubated with 80 μM of EGCG. Results are mean ± SD of *n* = 3. Note that the similar CID curves of YX-I-1 and EGCG suggest they bind to wt hIAPP with similar affinity, but do not indicate that the two molecules inhibit hIAPP aggregation through a similar mechanism of action. **d** The fluorescence emission spectra of 5 μM wt hIAPP in the absence or presence of concentrations of YX-I-1 ranging from 10 μM to 150 μM. **e** Schematic illustration of the aggregation mechanism of hIAPP in the presence of YX-I-1 or YX-A-1, showing the effect of each ligand on the individual microscopic events of aggregation. In the presence of inhibitor, primary and secondary nucleation pathways and elongation are suppressed, through interaction with peptide monomers. By contrast, the accelerator interacts predominantly with oligomeric species formed in the lag phase via primary nucleation pathways, and accelerates the aggregation of hIAPP. Native mass spectrometry experiments were performed more than three times (biological replicates). The fluorescence quenching experiments were repeated twice with different batches of the peptides.

To probe the nature of the interactions of YX-I-1 and YX-A-1 with wt hIAPP in molecular detail, nESI-MS was employed. This technique has been widely used for the characterisation of amyloid precursors and ligand interactions^64,73^. The nESI-mass spectra of wt hIAPP alone or in the presence of YX-I-1 are shown in Fig. 6a (i,ii) (Supplementary Fig. 13a). In the absence of small molecules, species spanning monomer, dimer and trimer were detected, that are co-populated in multiple charge states, with the triply charged monomer (monomer^3+^) ion dominating the spectrum (Fig. 6a (i)). WT hIAPP was incubated with different concentrations of YX-I-1 (molar ratio of peptide to inhibitor of 1:1, 1:3, 1:5 or 1:7). The resulting spectra show that YX-I-1 indeed binds monomeric wt hIAPP (preferentially to the 3+ ion), but does not bind to the dimers and trimers detected in this experiment (Fig. 6a (ii) and Supplementary Fig. 13a), consistent with the results presented above (although weak binding to lowly populated higher-order species not detected by nESI-MS cannot be ruled out). In addition, unlike the known colloidal inhibitor congo red^64^, no higher-order self-assembly of YX-I-1 was detected by nESI-MS, further supporting the DLS and NMR data that the small molecule does not self-associate into high-order particles. YX-A-1 was also found to bind monomeric wt hIAPP as judged by nESI-MS, although a smaller adduct peak was observed compared with that obtained with YX-I-1, suggestive of weaker binding (Fig. 6a (iii) and Supplementary Fig. 13b). Consistent with this supposition, combining wt hIAPP with an equimolar mixture of YX-I-1 and YX-A-1 (molar ratio: wt hIAPP:YX-I-1:YX-A-1 = 1:2.5:2.5), resulted in a higher ion intensity (~1.8-fold) for the inhibitor-bound state (Fig. 6a (iv)), and collision-induced dissociation (CID) experiments showed that dissociation of YX-A-1 occurred at a significantly lower voltage than for YX-I-1 (dissociation midpoints of 16.5 ± 0.1 Vand 12.4 ± 0.1 V, respectively (Fig. 6b, c). The binding of YX-A-1 to wt hIAPP monomers therefore must be too weak, too transient, or at a location that does not affect the rates of secondary nucleation and elongation. A control experiment using EGCG, which has previously been shown to bind to monomeric wt hIAPP using nESI-MS and to retard aggregation^73^, resulted in similar gas-phase stability as YX-I-1, consistent with the effectiveness of YX-I-1 as an inhibitor of wt hIAPP aggregation (Fig. 6c).

To map potential residues involved in the binding of YX-I-1 to monomeric IAPP, 2D SOFAST-HMQC NMR spectra of ^15^N-labeled IAPP-COOH in the presence of 2% (v/v) DMSO alone or YX-I-1 were obtained (Supplementary Fig. 14a). The spectra revealed small, but significant, chemical shift perturbations (CSPs) of several main-chain NHs in the presence of the small molecule, consistent with a weak binding interaction of the small molecule with the intrinsically disordered peptide ensemble present in the NMR sample^72,74^. Further quantification of the CSPs shows that NHs with the largest chemical shift differences include residues in the regions 10–14, 17–20 and 23–28 and the C-terminal Tyr37 (Supplementary Fig. 14b). The interactions with residues spanning V17 to S20 may rationalise, at least in part, the inability of YX-I-1 to inhibit the aggregation of S20G. In contrast to YX-I-1, no significant chemical shift perturbations of IAPP-COOH were observed in the presence of YX-A-1 in ^1^H-^15^N backbone NMR experiments (Supplementary Fig. 14c, d), further supporting the view that its function in accelerating aggregation is mediated by binding to rare species formed in the lag time of assembly. We also generated ^15^N-labelled S20G and acquired NMR spectra of the peptide in the presence or absence of YX-I-1 or YX-A-1. The ^1^H-^15^N HMQC spectrum of S20G containing YX-I-1 shows no major CSPs, suggesting that the inhibitor binds weakly, at best, to monomeric S20G, consistent with its inability to inhibit aggregation (Supplementary Fig. 15a, b). Only very minor CSPs were observed in the presence of YX-A-1 (Supplementary Fig. 15c, d). The acceleration of S20G aggregation observed with YX-A-1 presumably results from binding to lowly populated oligomeric species and/or via weak binding to monomers that are not detected by ^1^H-^15^N HMQC experiments.

The NMR results presented above suggest that the intrinsic fluorescence of wt hIAPP may change in the presence of YX-I-1, since Tyr37 is the sole fluorophore in the sequence (Fig. 1a). Consistent with this supposition, the fluorescence emission intensity of Tyr37 decreases significantly when the peptide is titrated with YX-I-1 (Fig. 6d). This confirms that YX-I-1 binds to monomeric wt hIAPP, consistent with the kinetic, nESI-MS and NMR data presented above. Analysis of the dependence of the fluorescence intensity of hIAPP on the concentration of YX-I-1 using the Stern-Volmer equation (Eq. (1)) (Supplementary Fig. 16b), yields a *K*_SV_ of 1.34 × 10^4^ M^-1^ and associated bimolecular quenching rate constant (*k*_q_) of 1.3 × 10^13^ M^-1^s^-1^ (assuming a fluorescence lifetime of tyrosine (τ_0_) of ~10^−9^ s (refs. ^75,76^)) (Supplementary Table 2). Since *k*_q_ exceeds substantially the maximum scattering collision quenching rate constant (~2 × 10^10^ ref. ^77^), a static quenching mechanism is likely. Accordingly, the *K*_d_ and binding stoichiometry of YX-I-1 binding to hIAPP could be determined using the modified Stern-Volmer equation (see Methods), yielding values of 32 μM and ~1:1, respectively (Supplementary Table 2). The same analysis was carried out for the accelerator YX-A-1 and EGCG, yielding a *K*_d_ for YX-A-1 and EGCG of ~280 μM and 13 μM (Supplementary Fig. 16, Supplementary Table 2). Again, the weak binding affinity of the accelerator is consistent with the nESI-MS, CID-MS and NMR results.

We further characterised the interactions between wt hIAPP and the small molecules using surface plasmon resonance (SPR). N-terminally biotinylated monomeric wt hIAPP was generated (Supplementary Fig. 17a), and the aggregation kinetics of the modified peptide was determined in the absence or presence of YX-I-1 or YX-A-1. As shown in Supplementary Fig. 17b, inhibition by YX-I-1 and acceleration by YX-A-1 were maintained for the modified peptide. Biotinylated wt hIAPP was then immobilised on the surface of the streptavidin sensor chip and the small molecules flowed over the surface. The binding affinity and kinetic behaviour of the three molecules were very different. As expected and consistent with the data presented above, YX-A-1 did not bind to immobilised wt hIAPP yielding no significant response signal (Supplementary Fig. 18b). By contrast, YX-I-1 and EGCG showed a concentration-dependent response, consistent with the binding of the small molecules to the peptide (Supplementary Fig. 18a,c). Interestingly, the binding kinetics of YX-I-1 and EGCG are very different: YX-I-1 interacts reversibly with the peptide with the rapid association and dissociation phases; while EGCG bound to the peptide with the slower association and dissociation phases (Supplementary Fig. 18a, c). Fitting of the steady-state response values to a 1:1 interaction model yielded *K*_d_ values of ~240 μM and 40 μM for YX-I-1 and EGCG, respectively (Supplementary Fig. 18d). Compared with the *K*_d_ determined by fluorescence quenching, the values determined using SPR are weaker for both small molecules, indicating that immobilising the peptide via its N-terminus perturbs binding and results in an aberrantly high apparent *K*_d_.

## Discussion

Although substantial efforts have been devoted to study the aggregation kinetics of hIAPP, including natural and non-natural sequence variants (reviewed in ref. ^3^), the detailed molecular mechanism(s) of hIAPP self-assembly into amyloid remained unclear. Here, we applied a chemical kinetics approach to investigate the aggregation of wt hIAPP and its natural variant S20G linked to early-onset T2D. The results revealed that the aggregation of both peptides is best described by a model that includes primary nucleation, multi-step surface-catalysed secondary nucleation and elongation (Fig. 6e). For S20G, an increased rate of primary nucleation, secondary nucleation and elongation, and a lower half-saturation concentration was observed, rationalising its more aggressive aggregation in vitro and involvement in early-onset disease^22^.

Dissection of the macroscopic aggregation kinetics into the microscopic events revealed that primary nucleation is at least 10^8^-times slower than the secondary process. The ratio of *k*_n_/*k*_2_ describes the concentration above which secondary nucleation will produce more nuclei than primary nucleation^33^. For both hIAPP variants *k*_n_/*k*_2_ is ~2 nM, further supporting the conclusion that under the quiescent conditions of our experiments new aggregates will be generated predominantly through secondary nucleation. *k*_n_/*k*_2_ for Aβ42 and Aβ40 is 23 nM and 0.6 nM, respectively^33^, demonstrating the importance of secondary nucleation in all of these systems.

In the second part of our study, we report the discovery of two small-molecule modulators of wt hIAPP assembly identified by a combinatorial nESI-MS and ThT bioassay approach, one (YX-I-1) an inhibitor and the second (YX-A-1) an accelerator of wt hIAPP assembly. Both compounds have not been reported previously as modulators of any other biological targets and have more favourable drug-like properties (lack of PAINS properties) compared with previously identified modulators such as EGCG and other polyphenols^44,58–60^. YX-I-1 is also highly specific, inhibiting the aggregation of wt hIAPP, but not the closely related peptides S20G or Aβ42 (Fig. 4c) and reducing the rate constants of primary nucleation, secondary nucleation and fibril elongation, indicating that the small molecule most likely interacts with peptide monomers (Fig. 6e). Subsequent nESI-MS, fluorescence, SPR and NMR results confirm this conclusion, suggesting that the binding site involves residues in its most aggregation-prone region (i.e. residues 17–20), and involves the C-terminal Tyr37, rationalising the specificity of YX-I-1 for the wt hIAPP sequence. While we are able to characterise the interactions between YX-I-1 and monomeric wt hIAPP using multiple approaches and no binding was observed to hIAPP dimers and trimers using nESI-MS, we cannot rule out the possibility that the inhibitor could interact with rarely populated higher-order oligomers formed during aggregation. Due to the heterogeneity and transient nature of these species, it is extremely challenging to characterise these complex interactions experimentally with the current techniques.

The identification of accelerators of amyloid formation lags behind that of inhibitors, with only a handful of small molecules reported to date (only polymers for wt hIAPP) that accelerate aggregation^51,78,79^. Here we show that YX-A-1 is a potent accelerator of the aggregation of wt hIAPP and S20G (but not Aβ42) (Fig. 4c). Its ability to accelerate hIAPP aggregation at substoichiometric concentrations suggests that YX-A-1 binds to oligomeric species formed via primary nucleation in the lag phase of assembly (Fig. 6e), a finding endorsed by the inability of YX-A-1 to enhance the rate of seeded growth (Supplementary Fig. 12). This was further supported by the weak interaction of YX-A-1 and wt hIAPP characterised by nESI-MS, CID-MS, SPR and fluorescence quenching. Unfortunately, it was not possible to determine the steps in assembly affected by YX-A-1 more precisely using the chemical kinetics approach, since none of the models tested (models varying primary nucleation and elongation (*k*_+_*k*_n_) or secondary nucleation and elongation (*k*_+_*k*_2_)) yielded an acceptable fit. Since higher-order amyloid oligomers were not detected using nESI-MS, we were unable to identify the specific species that interact with the accelerator using this technique. Nonetheless, the finding that the accelerator also enhances the aggregation of S20G suggests that the assembly reactions of wt hIAPP and S20G share structural as well as kinetic similarities, despite the fact that the fibrils that ultimately form from the two peptides differ in the structural organisation of the protofilaments and in the underlying subunit structure^26^.

While both YX-I-1 and YX-A-1 significantly alter the aggregation kinetics of wt hIAPP under the conditions employed here, it is important to note that other components in vivo such as metal ions^80,81^, insulin^82^, and membranes^83^, can affect the aggregation of hIAPP. It will be interesting to explore how YX-I-1 and YX-A-1 affect hIAPP aggregation in the presence of those components in the future. Similarly, since cross-seeding is observed between hIAPP and Aβ^16^, rationalising the pathological link between AD and T2D^16,84^, the effect of YX-I-1 and YX-A-1 on co-aggregation or cross-seeding may help to illuminate the mechanisms of how and why different proteins co-aggregate in amyloidosis.

In summary, the study presented here shows that the fibril formation mechanisms of Aβ and hIAPP share many features in common: they occur predominantly via surface-catalysed secondary nucleation and result in fibrils with similar structures^27^. The results also highlight how a single amino acid substitution can have a remarkable effect on fibril assembly: S20G forms fibrils that differ in their structural organisation compared with wt hIAPP, yet assembly occurs by a common kinetic mechanism that is again dominated by multi-step surface-catalysed secondary nucleation. Using small-molecule modulators of hIAPP assembly, we also reveal a striking specificity in the underlying mechanism of amyloid assembly in which YX-I-1 inhibits the assembly of wt hIAPP, but not S20G, while YX-A-1 accelerates aggregation of both peptides. Given that most modulators reported to date can change the aggregation of multiple protein/peptide targets (e.g. EGCG^44^), it is striking that both compounds modulate the aggregation of wt hIAPP with remarkable specificity, which reinforces the view that targeting distinct species formed by intrinsically disordered polypeptides or proteins which lack well-defined stable structures is possible^72,85^. Tailoring the rate of hIAPP aggregation by YX-I-1 and YX-A-1 will provide a powerful means to determine how altering the rate of assembly affects amyloid-associated cytotoxicity and/or the fibril structure(s) formed. More importantly, they may be used as promising starting points for the development of therapeutics towards T2D, a disease that currently affects more than 300 million individuals globally.

## Methods

### Preparation of wt hIAPP and S20G

WT hIAPP and S20G were synthesised using a Liberty Blue^TM^ automated microwave peptide synthesiser (CEM Microwave Technology) on a 0.1 mmol scale as reported previously^86,87^. 9-fluorenylmethyloxycarbonyl (Fmoc)-protected amino acids were used, and PAL-NovaSyn TG resin (Novabiochem^®^, Merck) was selected, allowing the generation of wt hIAPP/S20G with an amidated C-terminus. Three pseudoproline dipeptides (Fmoc-Ala-Thr(psiMe,MePro)-OH, Fmoc-Ser(tBu)-Ser(psiMe,MePro)-OH, and Fmoc-Leu-Ser(psiMe,Mepro)-OH, Merck) were used for the synthesis of Ala-8 and Thr-9, Ser-19 and Ser-20, and Leu-27 and Ser-28. All the residues and the three pseudoproline dipeptides were double coupled. The peptides were cleaved from the resin in a cleavage cocktail of trifluoroacetic acid (TFA) (9.4 mL), 3,6-dioxa-1,8-octanedithiol (DODT) (250 μL), H_2_O (250 μL) and triisopropylsilane (TIS) (100 μL). The mixture was stirred at room temperature for 3.5 h and then concentrated under a nitrogen stream. Subsequently, the crude peptide was precipitated in cold diethyl ether, followed by three washes with the same solvent. The peptide was then dissolved in a 50% (v/v) acetonitrile aqueous solution containing 0.1% (v/v) TFA and lyophilised. The peptide was then dissolved in 50% (v/v) DMSO aqueous solution to promote the formation of the internal disulfide bond between Cys-2 and Cys-7. The oxidised peptides were then purified by reverse-phase, high-performance liquid chromatography (HPLC) using a Kinetex^TM^ EVO C18 column (Phenomenex). The buffers used in the HPLC purification were acetonitrile with 0.1% (v/v) formic acid and H_2_O with 0.1% (v/v) formic acid. The masses of the purified peptides were confirmed by Electrospray Ionisation Mass Spectrometry (ESI-MS) as 3902.9 Da for hIAPP (expected = 3903.3 Da), and 3872.9 Da for S20G (expected = 3873.3 Da). The purity of the peptides was assessed by analytical HPLC and judged to be >95% pure. After purification peptides were lyophilised and stored at −20 °C until use.

### Initial screening and preparation of stock solutions of small molecules

Small molecules in the PPI library were generated by PPI-NET (https://ppi-net.org) and purchased from Asinex and ChemDiv and they were dissolved in DMSO with a stock concentration of 10 mM. In the nESI-MS screening a control protein (ubiquitin) was included in the solution to differentiate the specific binding and non-specific binding of the small molecules to wt hIAPP. Ligand screening using the ThT fluorescence was performed in 100 mM ammonium acetate pH 6.8 to match the nESI-MS conditions. Small molecules found to modulate the aggregation of wt hIAPP were then further evaluated in 25 mM sodium phosphate buffer, pH 6.8 to confirm that their effect was independent of the choice of the buffer used (Supplementary Fig. 5, full details of the screening experiments will be described elsewhere).

YX-I-1 and YX-A-1 were purchased from Asinex. 25 mM stock solutions were prepared in 100% DMSO and stored at −20 ^o^C for future use. The two compounds were characterised by ^1^H-NMR, ^13^C-NMR and HRMS (Supplementary Note 1).

### Preparation of wt hIAPP and S20G for kinetic analysis of aggregation

Low concentrations of preformed aggregates can significantly change the aggregation kinetics of hIAPP^29^. To remove any preformed small aggregates and ensure that the peptides were monomeric before commencing fibril formation assays, the peptides were dissolved in 100% hexafluorisopropanol (HFIP) at a concentration of 1 mg/mL and incubated at 4 °C for 24 h. The solution was then filtered through a 0.22 μm PDVF filter (Millex-GV, Merck) and the filtrate was aliquoted into 1.5 mL Eppendorf tubes. The solvent was dried over a gentle stream of nitrogen gas to form a film of peptide around the wall of the tubes. Samples were freeze-dried for 48 h. Peptides were stored at −20 °C and used within one month.

Tubes containing peptides were allowed to reach room temperature before opening. To make the peptide stock, filtrated pure water (kept on ice before use) was added into the tube. The absorbance of the solution was measured at 280 nm and the peptide concentration calculated using an extinction coefficient of 1620 M^−1^cm^−1^. The obtained peptide stock was diluted into 34.7 mM sodium phosphate buffer (pH 6.8) to the desired concentration, followed by the addition of DMSO or small-molecule stock. The mixture was then supplemented with 50 µM ThT from a 5 mM stock. 95 µL of the mixture was then pipetted into 96-well plate (half-area, clear bottom, Corning 3881) and sealed clear sealing film (SealPlate^TM^). The final buffer conditions were 25 mM sodium phosphate, pH 6.8 with 2% (v/v) DMSO. All samples were prepared on ice and care was taken when pipetting samples in order to avoid air bubbles. Control experiments using pyrene fluorescence showed the absence of micellar-like aggregates at all peptide concentrations studied here under the experimental conditions employed^88^.

For seeding assays, preformed fibril seeds were prepared by incubating 32 μM wt hIAPP or S20G under the same conditions as above. To create fibril seeds, fibrils were collected after the ThT kinetics reached a plateau (usually 24 h), and collected by centrifugation at 16,300 × *g*, 4 °C for 20 min. The supernatant was carefully removed. The amyloid fibrils were resuspended in the same buffer as the aggregation assay and sonicated in an ultrasonic water bath (Untrawave Ltd. Cardiff) for 10 s. The fibril seeds were mixed with samples containing 10 μM wt hIAPP or S20G monomers, and 95 µL of the mixture was then pipetted into 96-well plate (half-area, clear bottom, Corning 3881, sealed as above). The final seed concentrations were 0.063%, 0.25%, 1%, 2.5%, 5%, 10%, 20% or 30% (v/v).

For determining the effect of sonicating hIAPP fibrils on hIAPP aggregation, the fibrils of wt hIAPP and S20G were generated in 25 mM sodium phosphate buffer (pH 6.8) at 30 °C, quiescently, and they were then collected by centrifugation at 16,300 × *g*, 4 °C for 20 min. The supernatant was carefully removed. The fibril pellet was resuspended in the same buffer as the aggregation assay and separated into two fractions. One fraction was sonicated in an ultrasonic water bath (Untrawave Ltd. Cardiff) for 2 min; the other was kept in ice without sonication. The fibril seeds were mixed with samples containing 10 μM wt hIAPP or S20G monomers, and 95 µL of the mixture was then pipetted into 96-well plate (half-area, clear bottom, Corning 3881, sealed as above). The final seed concentrations were 5%, 20% or 30% (v/v) for wt hIAPP and 20%, 30% or 50% (v/v) for S20G.

### ThT fluorescence kinetic assays

Each 96-well plate was loaded with a sample, immediately sealed with clear sealing film (SealPlate^TM^) and incubated in a plate reader (CLARIOstar or FLUOstar Omega, BMG Labtech) at 30 °C quiescently. The ThT fluorescence intensity was measured through the bottom of the plate, using excitation and emission filters of 440 and 480 nm, respectively. Each experiment was performed at least twice with three replicates each time using different batches of the peptides.

### Aggregation kinetics of Aβ42

The aggregation kinetics of Aβ42 was performed according to the previously described protocol^70^. Briefly, to ensure that the aggregation kinetics is initiated from a monomeric state, lyophilised recombinant Aβ42 (synthesised and purified in-house) was dissolved in 6 M GuHCl buffer and passed through a size exclusion column (Superdex 75 10/300 GL column, GE Healthcare). The obtained monomer solution was then immediately diluted with 20 mM sodium phosphate buffer, pH 8.0 to the 3 µM peptide concentration, followed by the addition of DMSO or small-molecule stock. The mixture was then supplemented with 20 µM ThT from a 2 mM stock. 95 µL of the mixture was pipetted into the 96-well plate (half-area, clear bottom, Corning 3881, sealed as above). The final buffer conditions were 20 mM sodium phosphate, 200 μM EDTA, pH 8.0, 1% (v/v) DMSO. The 96-well plate was sealed with clear sealing film (as above) and incubated in a plate reader (CLARIOstar, BMG Labtech) at 37 °C quiescently. The ThT fluorescence intensity was measured through the bottom of the plate, using excitation and emission filters of 440 and 480 nm, respectively.

### Transmission EM

Ten microlitres samples from the ThT plates were placed onto glow-discharged carbon-coated copper grids for 1 min, and the excess liquid was blotted away. The grids were washed with 10 μL of pure water twice, followed by another wash with 1% (w/v) uranyl acetate. Another 10 μL 1% (w/v) uranyl acetate was added to stain the grids. The TEM images were acquired on a JEM-1400 (JEOL Ltd) transmission electron microscope in the Astbury Biostructure Laboratory.

### Theoretical analysis by AmyloFit

The t_50_ of hIAPP aggregation kinetics was generated by the online platform AmyloFit^40^. The t_50_ is the time point at which the fluorescence intensity reaches half of the maximum fluorescence intensity between the baseline and the plateau. The global fitting of kinetic models based on integrated rate laws for the amyloid fibril growth was used to generate the prediction curves to describe the experimental ThT kinetic data^40^. The combined rate constants (*k*_+_*k*_n_ and *k*_+_*k*_2_) and the half-saturation of secondary nucleation (√K_M_) of hIAPP fibril formation were obtained from global fitting of the experimental kinetics by using the online platform, AmyloFit^40^. For quantitative kinetic analysis of wt hIAPP aggregation in the presence of inhibitor YX-I-1, the same model determined for the concentration-dependent aggregation kinetics of wt hIAPP in the absence of modulators was used, including primary nucleation, multi-step secondary nucleation and elongation. The reaction orders for primary (n_c_) and secondary nucleation (n_2_) were fixed to 2. All the parameters constrained to a global constant, except the single parameter (*k*_+_*k*_n_, *k*_+_*k*_2_ or *k*_+_) which was set to fit. To ensure convergence, the number of basin hops was set to 10 for all the global fits, as described previously^40^.

### Solubility of small molecules and self-assembly propensity of the two modulators assessed by DLS and 1D-^1^H-NMR

The solubility of YX-I-1 and YX-A-1 was determined by a UV-based filtration assay^89^. It includes two main steps: (i) preparation of compound calibration curve and (ii) determination of compound solubility. The standards were made in an 80:20 (v/v) buffer (25 mM sodium phosphate, pH 6.8): acetonitrile solution to ensure complete compound solubility. The percentage of DMSO was maintained at 2% (v/v). To generate the calibration curves, samples with final concentrations of 500, 200, 50, 12.5, 3.13 or 0 μM small molecules were prepared and pipetted into a 96-well polypropylene, 2.4 mL deep-well plate. The plate was then covered with a polystyrene lid and shaken at 300 rpm for 30 min at room temperature. After shaking, 200 μL was transferred into a 96-well disposable UV analysis plate and the OD scanned at 10 nm increments from 250 to 500 nm. The wavelength (>270 nm) with maximum absorbance was selected to generate the calibration curve by plotting the absorbance versus the compound concentration.

One hundred ninety-six microlitres of 25 mM sodium phosphate buffer (pH 6.8) was dispensed into a MultiScreen^TM^ solubility filter plate (Millipore), followed by the addition of 4 μL of 25 mM compound stocks (in DMSO). The plate was sealed by parafilm and shaken at 300 rpm for 1.5 h at room temperature. A vacuum was then applied to the plate to retrieve the filtrate. One hundred sixty microlitres of the filtrate was transferred into a 96-well disposable UV analysis plate. Forty microlitres of acetonitrile was added and mixed thoroughly with a pipette. The plate was placed in a UV spectrometer and scanned at 10 nm increments from 250 to 500 nm. The wavelength used to generate the calibration curves was used to determine the solubility. All experiments were performed in triplicate.

For the DLS experiment, DMSO or small molecule stocks were diluted in 25 mM sodium phosphate buffer (pH 6.8). The final concentrations for YX-I-1 and YX-A-1 were 50 μM or 10 μM, respectively. The percentage of DMSO was maintained at 2% (v/v). The samples were analysed by batch-mode DLS using a Wyatt miniDawn TREOS system, equipped with an additional quasi-elastic light scattering (QELS-synonym for DLS) detector. The buffer baseline was recorded for 5 min in the Astra 6.1 software, followed by the injection of 300 μL of the solution. The data were collected for 5 min, prior to the injection of 1 mL buffer. Another five-minute of buffer baseline was recorded. The DLS data were analysed in Astra 6.1 software.

For the 1D-^1^H-NMR experiment, various small molecule stocks were diluted in 25 mM sodium phosphate buffer (pH 6.8) to achieve the required final concentrations. The percentage of DMSO was maintained at 2% (v/v). The samples were recorded by Bruker Ascend Aeon^TM^ 950 MHz spectrometer equipped with a TCI-cryoprobe (3 mm). Two hundred fifty-six scans and a recycling delay of 1 second were used to acquire the 1H-^1^D spectra. Data were processed and analysed on Topspin 4.03 software (Bruker).

### nESI-MS analysis

Polypeptide samples with a final concentration of 16 μM were prepared in 100 mM aqueous ammonium acetate buffer (pH 6.8). Small-molecule stocks in 100% DMSO were diluted into the buffer solution to achieve the required final concentrations (molar ratio of wt hIAPP and YX-I-1: 1:1, 1:3, 1:5 and 1:7; molar ratio of wt hIAPP and YX-A-1: 1:0.5, 1:1 and 1:2). The percentage of DMSO in the solution was 2% (v/v) for all of the samples. Native ESI-MS analysis was performed on a Synapt G1 HDMS instrument (Waters Corp., Wilmslow, UK) equipped with a Triversa NanoMate (Advion Biosciences) automated nano-ESI interface.

All the samples were analysed using positive ionisation ESI with a capillary voltage of 1.6 kV and a N_2_ nebulising gas pressure of 0.5 psi (34.5 mbar). The following instrumental parameters were used: source temperature 30 °C; sampling cone 30 V; backing pressure 2.3 mbar; extraction cone 1 V; trap collision energy 5 V; trap DC bias 25 V; transfer collision energy 2 V. The system was calibrated with NaI cluster ions from a 2 μg/μL 50:50 2-propanol:water solution. Data were acquired over the *m/z* range of 200–4000 and processed by using MassLynx V4.1 supplied with the mass spectrometer.

CID-MS/MS experiments were conducted in the trap cell of the Synapt G1 mass spectrometer with argon gas. The ligand-bound hIAPP monomer complexes were isolated by the quadrupole analyser and the collision energy was applied increasingly to the trap cell from 5 to 35 V until the wt hIAPP-ligand complex completely dissociated. All the mass spectrometry experiments were repeated at least three times.

### 2D ^1^H-^15^N interaction analysis

For NMR experiments, recombinant ^15^N-labeled wt IAPP and S20G with a C-terminal carboxylic acid were used and the peptide was prepared as described previously^90^. Lyophilised hIAPP/S20G-COOH was monomerised using the same method as described above. NMR samples were prepared by diluting the peptide stock in 25 mM sodium phosphate buffer, pH 6.8 at a concentration of 20 μM containing 8% (v/v) D_2_O. DMSO (final concentration 2% (v/v)) or small molecule (final concentration: 100 µM for YX-I-1 and 20 µM for YX-A-1) was then added. 2D ^1^H-^15^N NMR spectra were acquired on a Bruker Ascend Aeon^TM^ 950 MHz spectrometer equipped with a TCI-cryoprobe (3 mm). The probe temperature was set to 15 °C. Chemical shift perturbation (CSP) was quantified by using ^1^H-^15^N resonances in the ^1^H-^15^N SOFAST-HMQC spectra. Amide CSPs were calculated as ^1^H^15^N-CSP = $\sqrt{\left[5* {\left(\Delta \delta H\right)}^{2}\right]+{(\triangle \delta N)}^{2}}$. All NMR spectra were processed and analysed using NMRPipe^91^ and ccpNMR-Analysis^92^ software, respectively. The ^1^H-^15^N resonance assignment of hIAPP was transferred from data previously reported in the Biological Magnetic Resonance Data Bank (access number: 26706). Control experiments showed that YX-I-1 and YX-A-1 retain the ability to modulate the aggregation kinetics of IAPP-COOH (i.e. lacking the native C-terminal amide) and the formation of fibrils at the end of the reaction (Supplementary Fig. 14e–g).

### Fluorescence quenching of wt hIAPP by YX-I-1, YX-A-1 and EGCG

DMSO stocks of YX-I-1, YX-A-1 or EGCG were diluted into 25 mM sodium phosphate buffer (pH 6.8) in the absence or presence of 5 μM wt hIAPP. The final percentage of DMSO was maintained at 2% (v/v). Tyr fluorescence was excited at 276 nm and the fluorescence emission spectrum (300 nm to 370 nm for YX-I-1 and 300 nm to 330 nm for YX-A-1 and EGCG) of the DMSO blank, and wt hIAPP in the absence or presence of small molecule were measured using a Quantum Master Fluorimeter (Turret 400^TM^, Photon Technology) controlled by FelixGX software v4.3 with excitation and emission slits of 5 nm. The final emission spectra were obtained by subtracting the emission spectra of DMSO or small molecule alone from the corresponding emission spectra of DMSO or small molecule with wt hIAPP.

To study the quenching mechanism of wt hIAPP by each small molecule, the data were analysed using the Stern-Volmer equation^77^,

1 $$\frac{{{{\mathrm{F}}}}_0}{{{\mathrm{F}}}}=1+{k}_{{{\mathrm{q}}}}{{\tau} _{0}}\left[{{{\rm{Q}}}}\right]=1+{{K}_{{{\mathrm{sv}}}}}[{{{\rm{Q}}}}]$$

where F_0_ is the fluorescence intensity of wt hIAPP alone, F is the fluorescence intensity of wt hIAPP in the presence of different concentrations of small molecule, [Q] is the concentration of a small molecule, *K*_sv_ is the Stern−Volmer constant, *k*_q_ is the quenching rate constant, and τ_0_ is the average lifetime of the fluorophore (here the lifetime for tyrosine fluorescence (assumed to be 10^−9^ s (refs. ^75,76^)).

To calculate the quenching rate constant *k*_q_, the following equation was used,

2 $${k}_{q}=\frac{{K}_{{{\mathrm{sv}}}}}{{\tau }_{0}}$$

The fluorescence quenching data of wt hIAPP were used to determine the binding constant (*K*_b_) and the number of binding sites (n) per wt hIAPP molecule using,

3 $${{{\mathrm{Log}}}}\left[\frac{{{\mathrm F}_{0}}-{\rm F}}{{{{{\mathrm{F}}}}}}\right]={\rm Log}\,K_{\rm b}+{\rm nLog}[{\rm Q}]$$

where F_0_, F and [Q] are the same as those in the Stern-Volmer equation.

### Surface plasmon resonance

The kinetics of the interaction between wt hIAPP and small molecules (YX-I-1, YX-A-1 and EGCG) was analysed by SPR on Biacore T200 (Cytiva) at 25 °C. N-terminally biotinylated monomeric wt hIAPP was generated by reacting wt hIAPP with NHS-PEG_4_-biotin (Thermo Scientific™). Singly modified wt hIAPP was obtained after the purification by HPLC (wt hIAPP contains a single N-terminal Lys) (Supplementary Fig. 17). This peptide was immobilised on a streptavidin sensor chip (Sensor Chip SA, Cytiva), and an untreated flow cell was used as the reference surface. The running buffer was 25 mM sodium phosphate, pH 6.8, 2% DMSO. DMSO control and various concentrations of small molecules were prepared. The solutions were injected for 60 s at 30 μL/min with a dissociation phase of 450 s. The process was performed twice for each compound. Response from the reference surface and a buffer (DMSO) injection over derivatised surfaces were subtracted from derivatised flow-cell data (BIA evaluation v2.0). The binding sensorgram data were analysed using Origin software 2019b and the steady-state plots for the binding of YX-I-1 or EGCG to wt hIAPP was analysed using GraphPad Prism 8.

### Interaction between small molecules and wt hIAPP fibrils

To test whether the small molecules are able to bind wt hIAPP fibrils, the fibrils (monomer concentration of 10 μM) were incubated with DMSO, 50 μM YX-I-1 or 10 μM YX-A-1 at r.t. for 5 min. Fibrils were spun down and the UV absorbance of the supernatant were recorded. To test the depolymerisation effect of the inhibitor and accelerator, wt hIAPP fibrils (monomer concentration of 10 μM) were incubated with 2% (v/v) DMSO alone, or 2% (v/v) DMSO containing YX-I-1 (50 μM) or YX-A-1 (10 μM) in 25 mM sodium phosphate buffer at 30 °C quiescently. The ThT fluorescence intensity was measured for 40 h in a plate reader (CLARIOstar, BMG Labtech).

### AFM sample preparation and imaging

The distributions of fibril height and fibril length were determined by atomic force microscopy (AFM). AFM was carried out in liquid in Peak Force tapping mode using a Bruker Multimode 8 AFM with a Nanoscope V controller using Bruker PeakForce-HIRS-SSB probes. A sample volume of 90 μL of fibrils formed in the presence of DMSO, YX-I-1 or YX-A-1 were added to freshly cleaved mica and allowed to bind to the surface for 1 hour before rinsing with buffer (50 mM sodium phosphate, 300 mM KCl, pH 7.5) via fluid exchange, maintaining the samples in a liquid environment by fluid. Heights and lengths of fibrils were measured either automatically using MATLAB to trace along the backbone of the fibril (https://github.com/George-R-Heath/Correlate-Filaments) or manually in ImageJ for densely packed overlapping fibrils.

### Reporting summary

Further information on research design is available in the Nature Research Reporting Summary linked to this article.

## Supplementary information

Supplementary Information

Reporting Summary

## Data Availability

The data that support this study are available from the corresponding authors upon reasonable request. All ThT kinetics, EM images, nESI-mass spectra, NMR, fluorescence quenching, UV absorbance, SPR data and AFM analysis are available at the University of Leeds Data Repository: 10.5518/1000. Source data are provided with this paper.

## Source data

Source Data
