# Supplementary material for: Tuning the rate of aggregation of hIAPP into amyloid using small-molecule modulators of assembly
Source: Nat Commun. 2022 Feb 24;13:1040. doi: 10.1038/s41467-022-28660-7 (PMC8873464; doi:10.1038/s41467-022-28660-7)

Supplementary information

Tuning the rate of aggregation of hIAPP into amyloid using small-molecule modulators of assembly

Yong Xu[1], Roberto Maya-Martinez[1], Nicolas Guthertz[1], George R. Heath[2], Iain W. Manfield[1], Alexander L. Breeze[1], Frank Sobott[1], Richard Foster[3*] and Sheena E. Radford[1*]

[1] Astbury Centre for Structural Molecular Biology, School of Molecular and Cellular Biology, Faculty of Biological Sciences, University of Leeds, LS2 9JT, United Kingdom

[2] Astbury Centre for Structural Molecular Biology, School of Physics & Astronomy, University of Leeds, Leeds, LS2 9JT, United Kingdom

[3] Astbury Centre for Structural Molecular Biology, School of Chemistry, University of Leeds, LS2 9JT, United Kingdom

* Corresponding authors: Richard Foster (r.foster@leeds.ac.uk) & Sheena E. Radford (S.E.Radford@leeds.ac.uk)

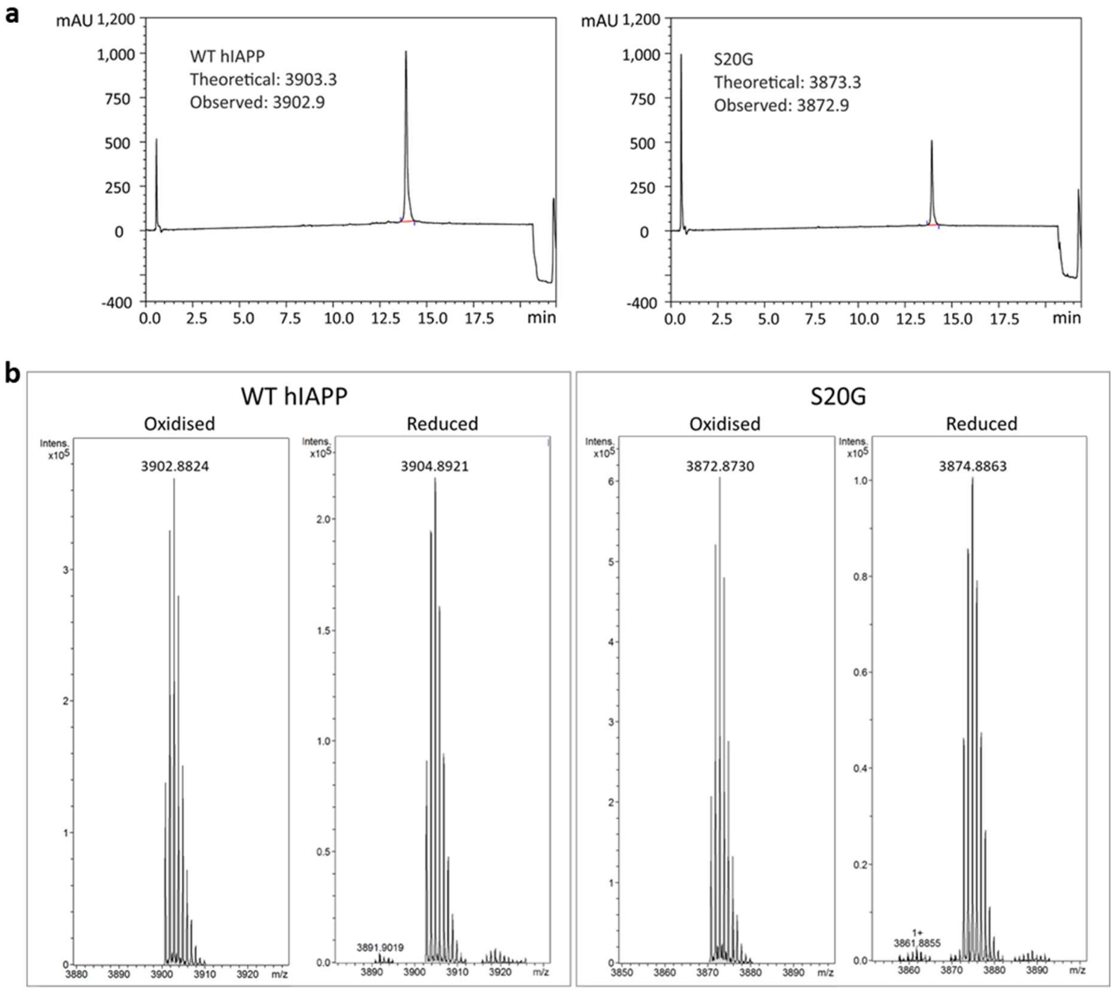

Supplementary Fig. 1. Characterisation of the in-house synthesised wt hIAPP and its S20G variant. (a) Analytical HPLC traces of wt hIAPP (left) and S20G (right). The expected and observed masses of the oxidised peptides (in Da) are shown. (b) ESI-mass spectra before (reduced) and after (oxidised) formation of the disulfide bridge between residues 2 and 7 for wt hIAPP (left panels) or S20G (right panels). The masses (Da) are shown above each spectrum.

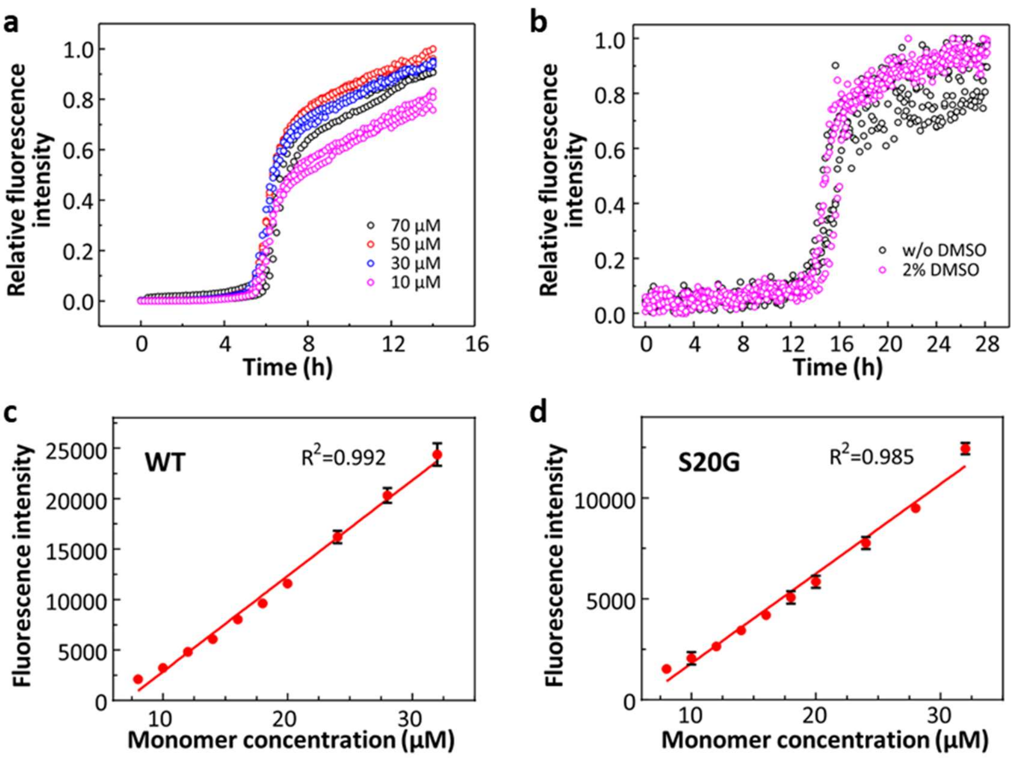

Supplementary Fig. 2. Control experiments required for detailed kinetic analysis of the aggregation kinetics of wt hIAPP and S20G. (a) Aggregation kinetics of wt hIAPP (32 μM) in the presence of different concentrations of ThT. (b) Addition of 2% (v/v) DMSO does not affect the aggregation kinetics of 10 µM wt hIAPP. (c&d) Plot of maximum ThT fluorescence intensity versus initial monomer concentration of (c) wt hIAPP and (d) S20G. The linear correlation observed in (c) and (d) confirm that the ThT fluorescence intensity scales linearly with the fibril mass, as shown for Aβ in previous studies[33-36]. All reactions were performed in 25 mM sodium phosphate buffer, pH 6.8, 30 °C, quiescently. Results are mean ± SD of n = 3.

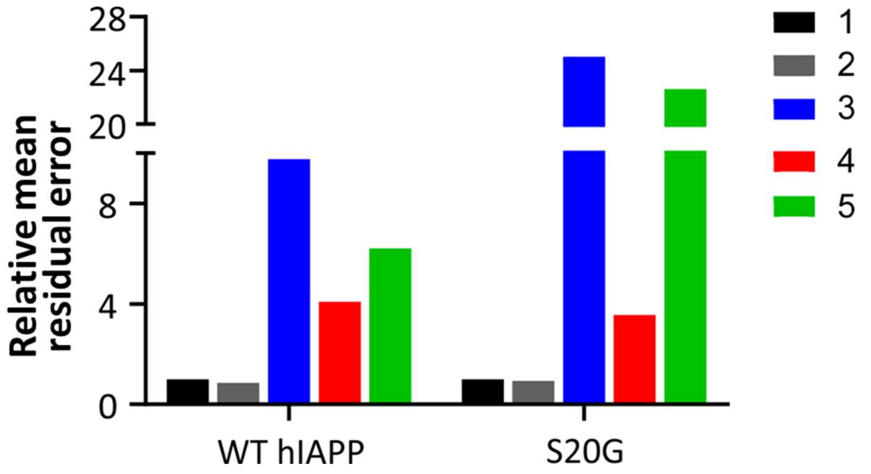

Supplementary Fig. 3. Mean residual error (MRE) of different models relative to the model including primary nucleation, elongation and multi-step secondary nucleation. MRE is a measure to compare the goodness of different fits[40]. 1 indicates the model that includes primary nucleation, elongation and multi-step secondary nucleation with fixed reaction orders (primary (n_c) and secondary nucleation (n_2) were fixed to 2); 2 indicates the model that includes primary nucleation, elongation and multi-step secondary nucleation with variable reaction orders (n_c and n_2). Under this model, the n_c and n_2 for WT hIAPP are 1.91 and 2.25, respectively, and the n_c and n_2 for S20G are 1.68 and 2.18, respectively, 3 includes the model that includes primary nucleation and elongation; 4 indicates the model that includes primary nucleation, fragmentation and elongation; 5 indicates the model that includes primary nucleation, secondary nucleation and elongation.

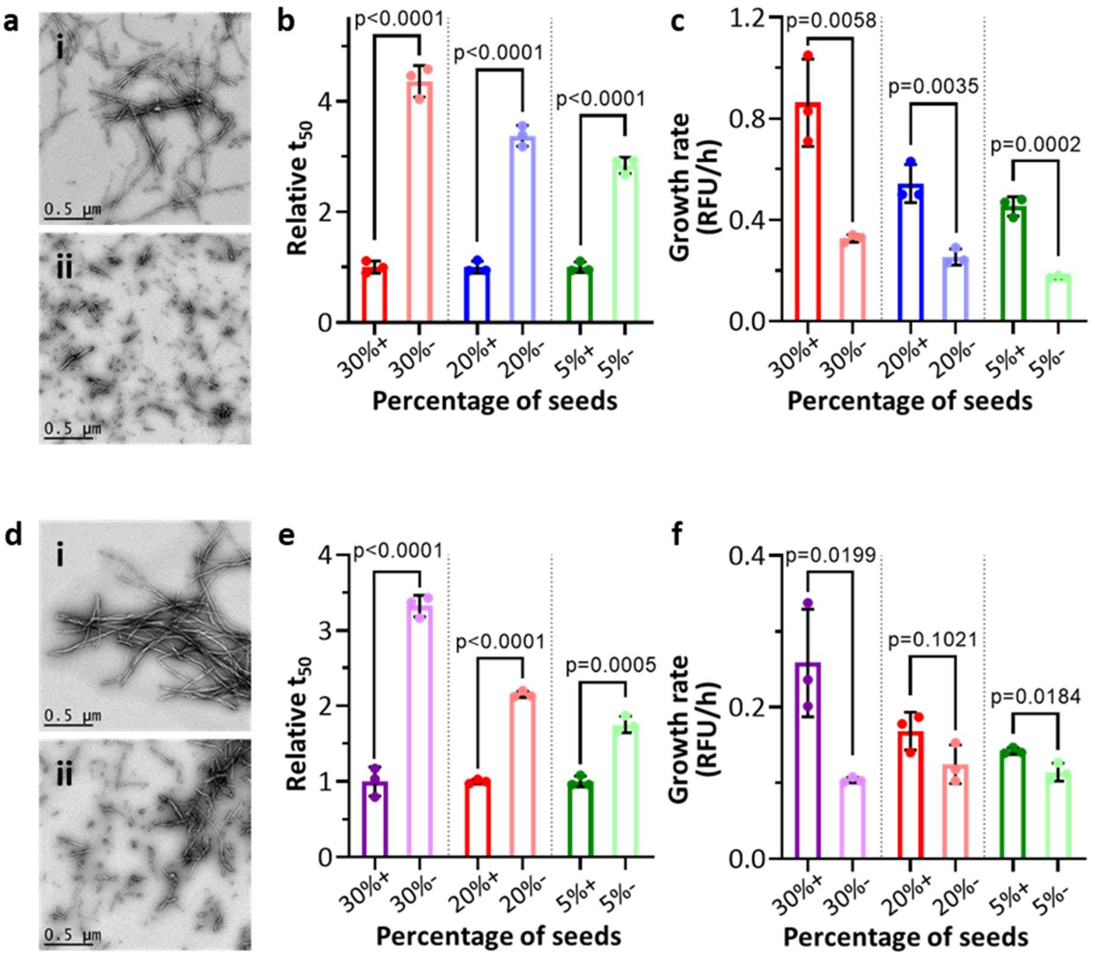

Supplementary Fig. 4. Effect of sonicating the hIAPP fibrils on hIAPP aggregation rate shows that elongation contributes significantly to fibril growth especially at high seed concentrations. (a) TEM images of wt hIAPP fibrils without (i) or with (ii) sonication. The scale bar is 0.5 µm. The TEM images are consistent in two biological replicates. (b) Relative half time (t_{50}) of aggregation of wt hIAPP in the presence of different percentage of fibril seeds with (+) or without (-) sonication. Relative t_{50} = $t_{50\ (\text{without sonication})}/t_{50\ (\text{with sonication})}$ at the corresponding seed concentration. (c) Growth rate (RFU (Relative Fluorescence Units)/h) of wt hIAPP aggregation in the presence of different percentage of seeds with (+) or without (-) sonication. (d) TEM images of S20G fibrils without (i) or with (ii) sonication. The scale bar is 0.5 µm. The TEM images are consistent in two biological replicates. (e) Relative half time (t_{50}) of aggregation of S20G in the presence of different percentage of fibril seeds with (+) or without (-) sonication. (f) Growth rate (RFU/h) of S20G aggregation in the presence of different percentage of seeds with (+) or without (-) sonication. Growth rate constant was determined by fitting a gradient to the linear part of the ThT kinetic curves using OriginPro software (OriginPro 2018b 64Bit). Each experiment was performed three technical replicates. Bars represent the mean and error bars show standard deviation (n = 3), with values for each replicate shown as points. P values were determined by t test (two tailed) using GraphPad Prism 8. Quantification of the t_{50} and growth rate showed statistically significant differences between the seeds with or without sonication ($p < 0.05$). The exact p values were shown in each figure.

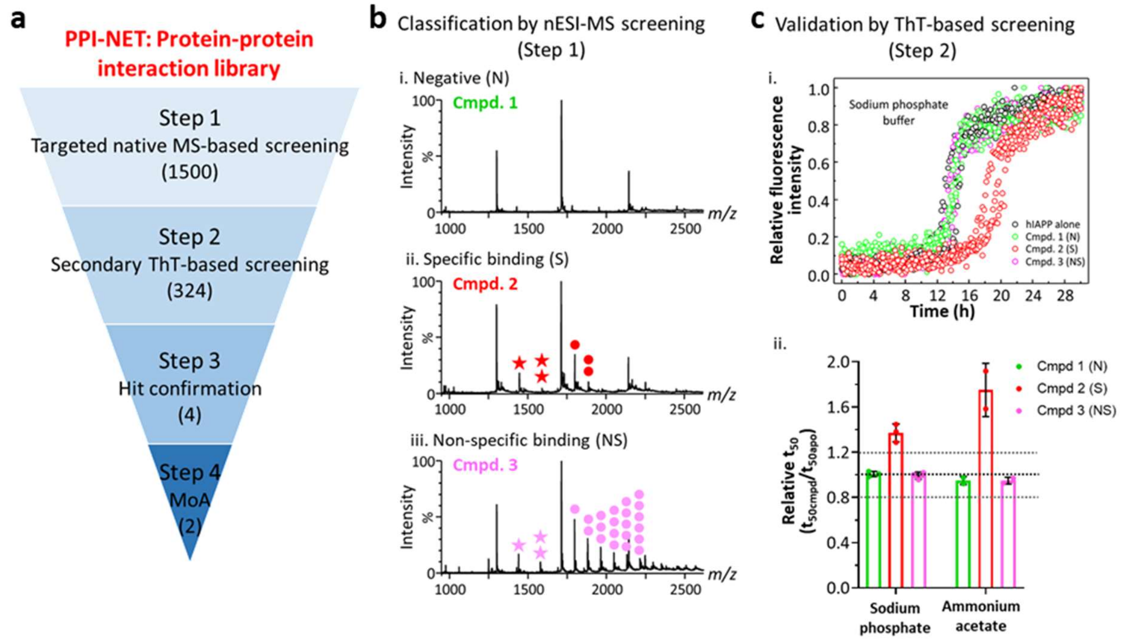

Supplementary Fig. 5. Schematic description of workflow for the small molecule modulator discovery targeting hIAPP aggregation. (a) Small molecule 'hits' were identified and prioritised by a hierarchical 4-step screening cascade. Step 1 is a primary nESI-MS screen of 1500 compounds from the protein-protein interaction (PPI) library. Step 2 is a secondary ThT-based screen with 324 compounds selected from the first step (including compounds with no binding, specific binding and non-specific binding to wt hIAPP). Four hits were confirmed based on satisfying two criteria: (1) the ability to bind to monomers/oligomers of wt hIAPP using nESI-MS and (2) significant alteration of the t_{50} of aggregation kinetics measured using ThT fluorescence. Two compounds were selected for detailed mode-of-action characterisation owing to their substantial effect on hIAPP aggregation (Step 4). (b) nESI-MS primary screening in Step 1 results in mass spectra showing different interactions between hIAPP or control protein (ubiquitin) and small molecules. These binding profiles are classified into three groups (i. negative, N; ii. specific, S; iii. non-specific, NS), which are represented by three small molecule examples (cmpd. 1, 2 and 3) from the PPI library. Molecules that do not bind to hIAPP are classified as negative (Supplementary Fig. 5b (i), charge state distributions from only hIAPP and control protein were observed in the native mass spectrum); molecules binding to IAPP and resulting in less than 3 copies of the small molecules bound to the control protein are classified as specific binding (Supplementary Fig. 5b (ii)); molecules binding to IAPP and resulting in more than 3 copies of the small molecules bound to the control protein are classified as non-specific binding (Supplementary Fig. 5b (iii)). Bound peaks are denoted with stars (peaks of hIAPP-small molecule complex) or circles (peaks of control protein-small molecule complex); the number of stars or circles represents the number of small molecules bound. (c) Small molecules selected from the nESI-MS primary screening were further evaluated by ThT-based secondary screening in two different buffers (ammonium acetate buffer and sodium phosphate buffer). The effect of cmpd. 1 (green), 2 (red) and 3 (pink) on hIAPP aggregation in sodium phosphate are shown (i). Relative t_{50} of hIAPP aggregation in the presence of the molecules in the two buffer systems was calculated (ii). Molar ratio of 1:5 wt IAPP:small molecule were used for the screen. Bars represent the mean and error bars show standard deviation (n = 3 for sodium phosphate buffer and n =2 for ammonium acetate buffer), with values for each replicate shown as points. Small molecules classified as negative (cmpd. 1) or non-specific binding (cmpd. 3) by nESI-MS

do not change the aggregation kinetics of hIAPP; while compounds classified as specific (cmpd. 2) by nESI-MS can alter the aggregation kinetics of hIAPP significantly. Compounds changing the t_{50} by more than 20% were selected for detailed analysis.

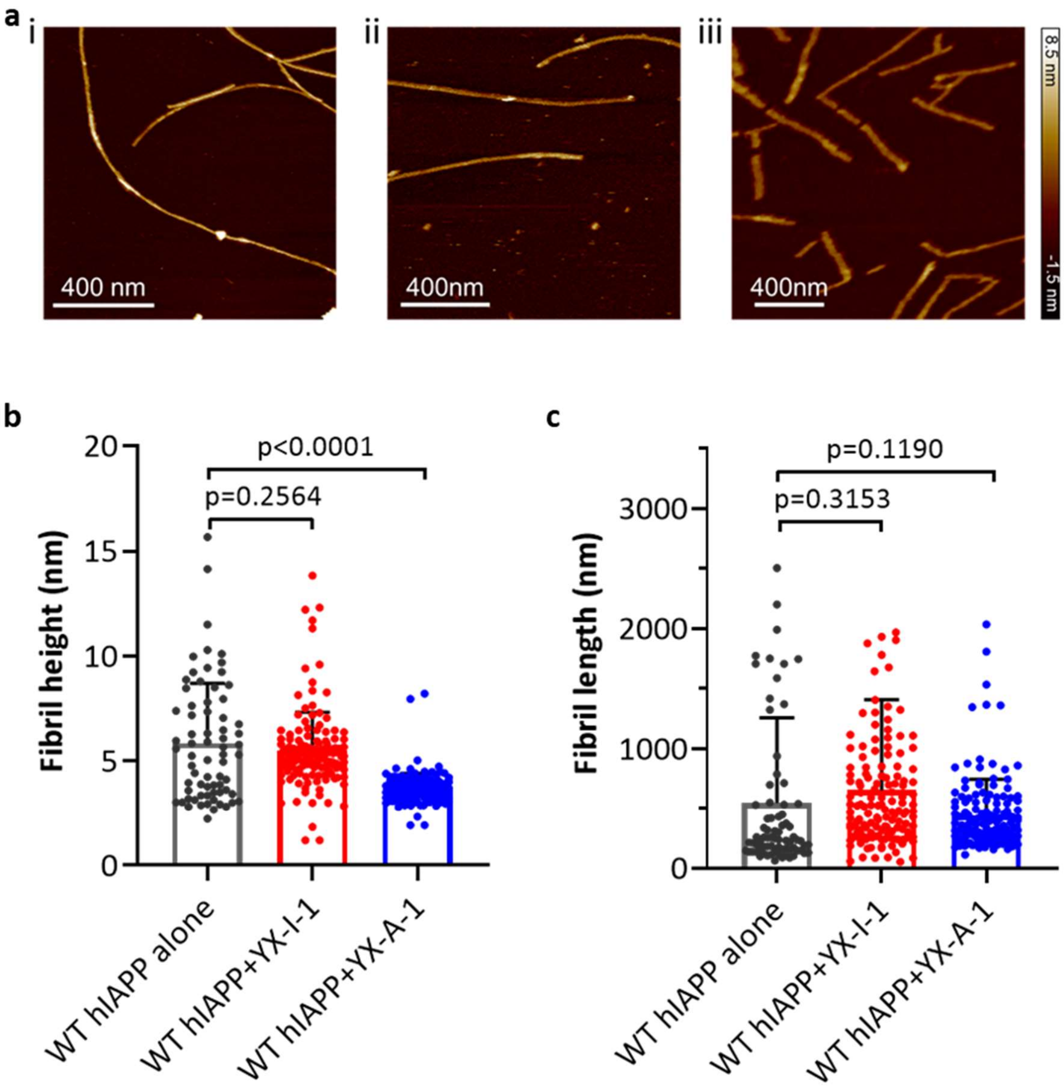

Supplementary Fig. 6. AFM characterization of wt hIAPP fibrils formed in the presence of DMSO, YX-I-1 or YX-A-1. (a) AFM analysis of wt hIAPP fibrils formed in the presence of DMSO (i), YX-I-1 (ii) or YX-A-1 (iii). Scale bars and their accompanying dimensions are indicated on each panel. (b) Distributions of heights of 10 µM wt hIAPP fibrils formed in the presence of DMSO (n=70), 50 µM YX-I-1 (n=143) or 10 µM YX-A-1 (n=143) by single molecule analyses of AFM images. The height of the columns indicated means. P values were determined by t test. Fibrils formed in the presence of YX-A-1 have lower fibril height (p<0.0001) than those formed in the presence of DMSO; while fibrils formed in the presence of YX-I-1 have similar fibril height as the DMSO-treated samples (p=0.2564). (c) Distributions of fibril length of 10 µM wt hIAPP fibrils formed in the presence of DMSO (n=70), 50 µM YX-I-1 (n=143) or 10 µM YX-A-1 (n=143) by single molecule analyses of AFM images. Bars represent the mean and error bars show standard deviation. P values were determined by t test (two tailed) using GraphPad Prism 8. Fibrils formed in the presence of YX-I-1 (p=0.3153) or YX-A-1 (p=0.1190) have similar fibril length as those formed in the presence of DMSO.

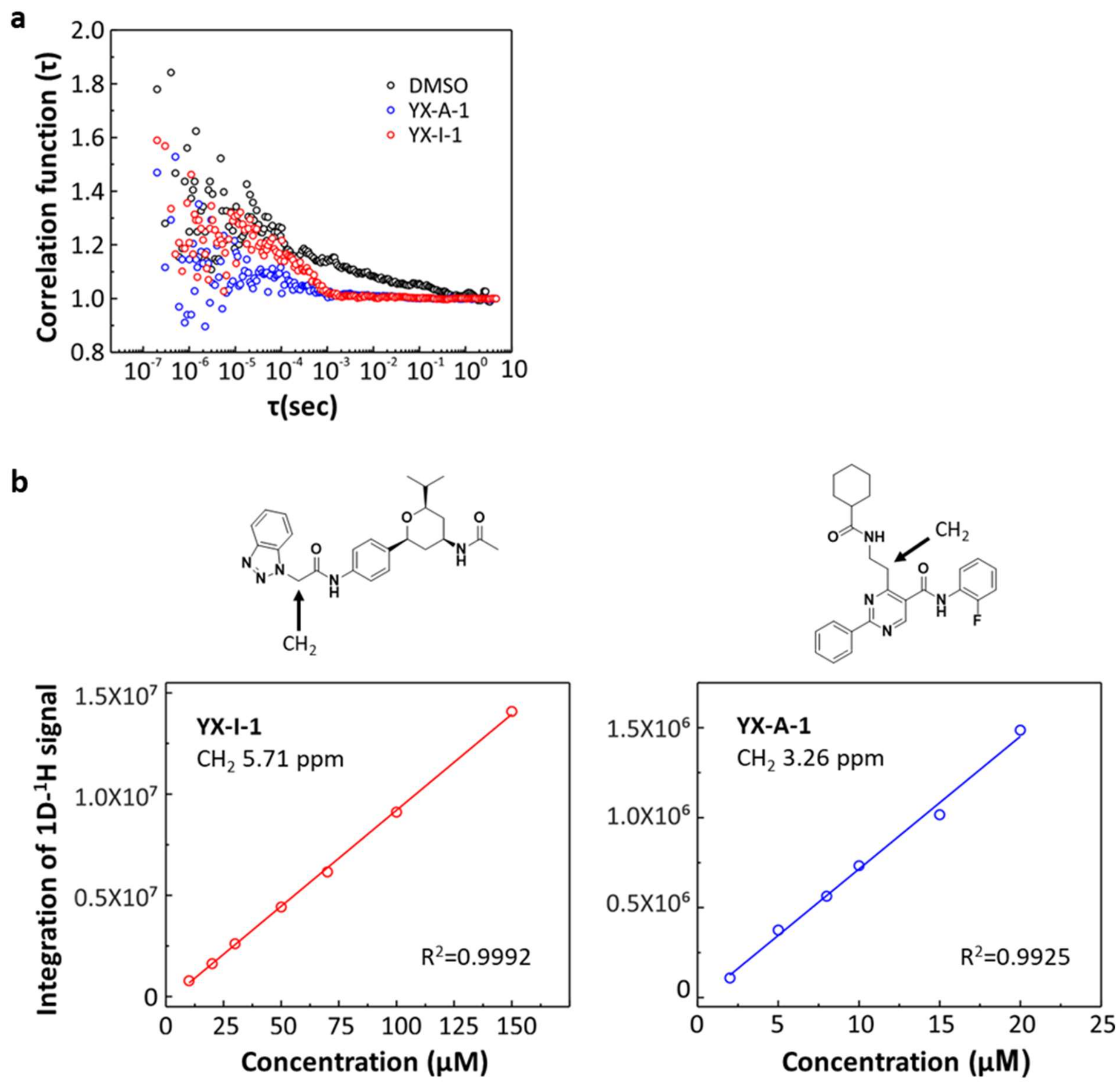

Supplementary Fig. 7. YX-I-1 and YX-A-1 do not self-assemble under the experimental conditions employed. (a) Autocorrelation function plot obtained by DLS analysis. DMSO control, 50 μM YX-I-1 and 10 μM YX-A-1 are shown in black, red and blue. All the samples were prepared in 25 mM sodium phosphate buffer (pH 6.8) with a final DMSO concentration of 2% (v/v). (b) Integration of 1D-^{1}H NMR signal of inhibitor (YX-I-1) and accelerator (YX-A-1) as a function of their concentration. Resonances at 5.71 ppm and 3.26 ppm were chosen for inhibitor and accelerator, respectively.

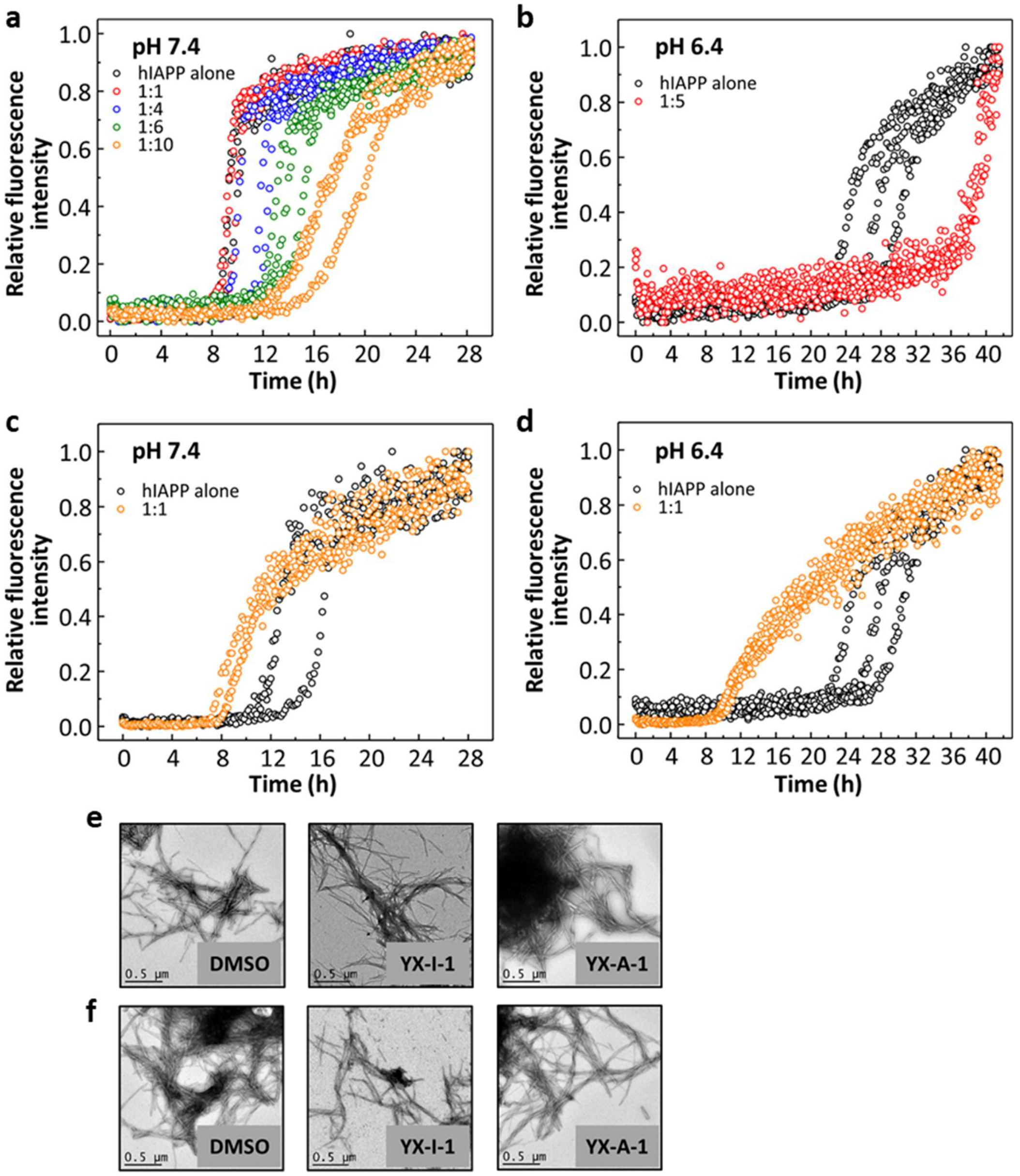

Supplementary Fig. 8. YX-I-1 and YX-A-1 modulate the aggregation kinetics of hIAPP at different pH values. (a) Dose-dependent inhibition of wt hIAPP aggregation by YX-I-1 at pH 7.4. Relative ThT fluorescence intensity of 10 μM wt hIAPP in the absence (black) or presence of a 1:1 (red), 1:4 (blue), 1:6 (green) or 1:10 (yellow) molar ratio of wt hIAPP to YX-I-1. (b) Inhibition of wt hIAPP aggregation by YX-I-1 at pH 6.4. Relative ThT fluorescence intensity of 10 μM wt hIAPP in the absence (black) or presence of a 1:5 (red) molar ratio of wt hIAPP to YX-I-1. (c) Acceleration of wt hIAPP aggregation by YX-A-1 at pH 7.4. Relative ThT fluorescence intensity of 10 μM wt hIAPP in the absence (black) or presence of a 1:1 (yellow) molar ratio of wt hIAPP to YX-A-1. (d) Acceleration of wt hIAPP aggregation by YX-A-1 at pH 6.4. Relative ThT fluorescence intensity of 10 μM wt hIAPP in the absence (black) or presence of a 1:1 (yellow) molar ratio of wt hIAPP to YX-A-1. All the ThT kinetic experiments were performed in triplicate. (e) TEM images of wt hIAPP incubated with DMSO, 60 μM YX-I-1 or 10 μM YX-A-1 at pH 7.4. (f) TEM images of wt hIAPP incubated with DMSO, 50 μM YX-I-1 or 10 μM YX-A-1 at pH 6.4. The scale bar is 0.5 μm. The TEM images are consistent in all three

experimental replicates. All reactions were performed in 25 mM sodium phosphate buffer containing 2% (v/v) DMSO at the indicated pH, 30 °C, quiescently.

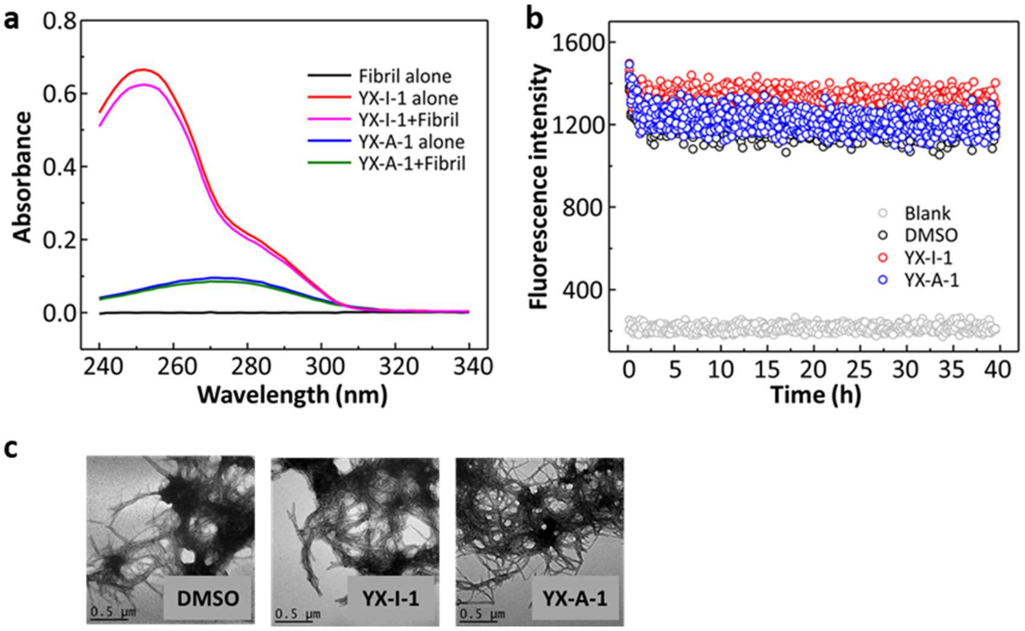

Supplementary Fig. 9. The effect of YX-I-1 and YX-A-1 on wt hIAPP fibril stability. (a) YX-I-1 and YX-A-1 do not bind wt hIAPP fibrils. WT hIAPP fibrils (monomer concentration of 10 µM) were incubated with 2% (v/v) DMSO alone, and 2% (v/v) DMSO containing 50 µM YX-I-1 or 10 µM YX-A-1. Fibrils were pelleted by centrifugation and the UV absorbance of the supernatant was recorded. (b) WT hIAPP fibrils (monomer concentration of 10 µM) were incubated with 2% (v/v) DMSO alone, or 2% (v/v) DMSO containing YX-I-1 (50 µM) or YX-A-1 (10 µM). The ThT fluorescence intensity remains unchanged in the presence of the small molecules, indicating that the two compounds do not deploymerise the fibrils, at least under the conditions used. (c) TEM images of wt hIAPP fibrils incubated with 2% (v/v) DMSO alone or 2% (v/v) DMSO containing 50 µM YX-I-1 or 10 µM YX-A-1. The scale bar is 0.5 µm. The TEM images are consistent in all three experimental replicates.

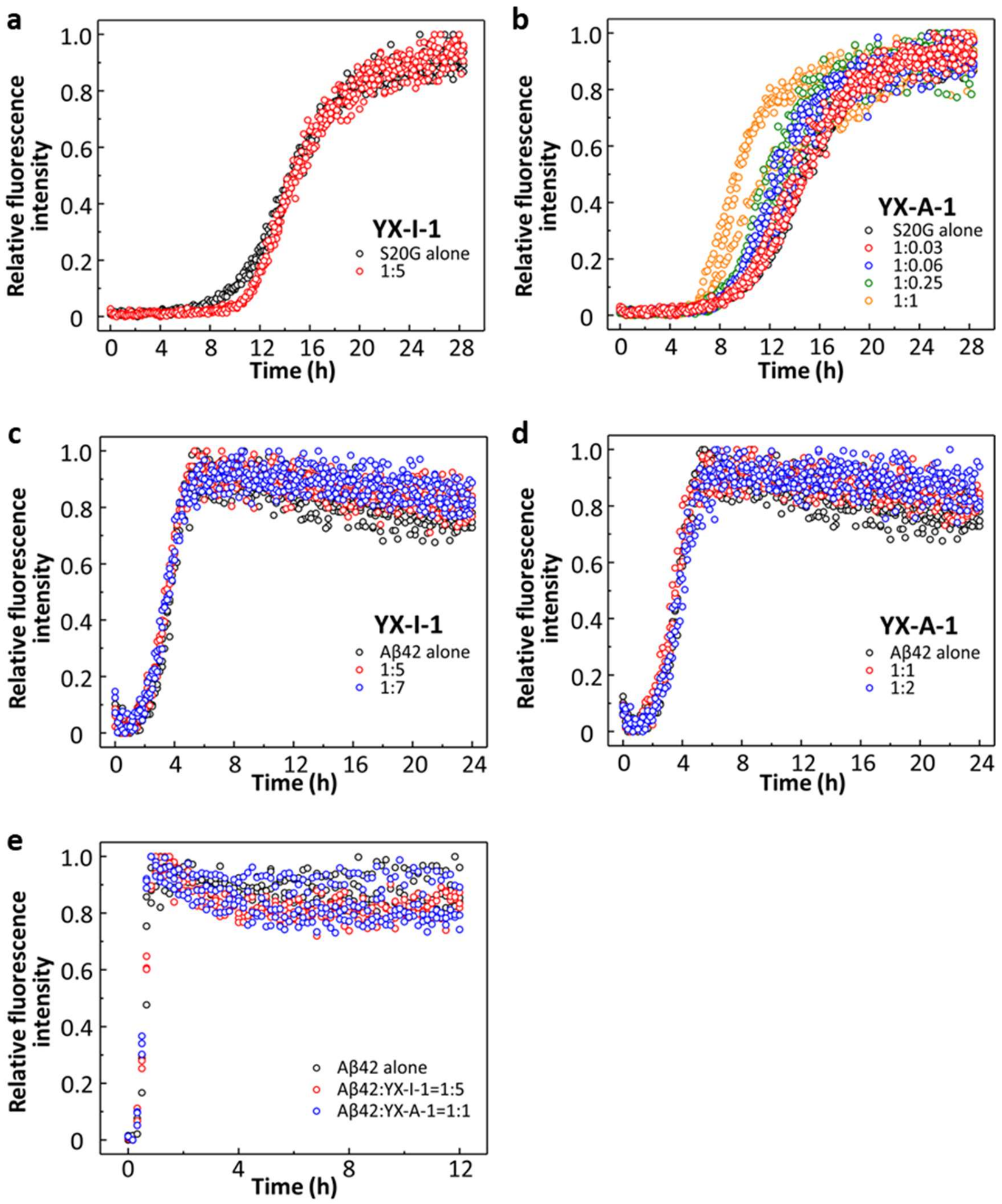

Supplementary Fig. 10. Effect of YX-I-1 and YX-A-1 on the aggregation kinetics of S20G and Aβ42. (a) Aggregation kinetics of 10 μM S20G in the presence of 0 μM or 50 μM of YX-I-1. (b) Aggregation kinetics of 10 μM S20G in the presence of 0 μM, 0.3 μM, 0.6 μM, 2.5 μM, or 10 μM of YX-A-1. All reactions with S20G were performed in 25 mM sodium phosphate buffer containing 2% (v/v) DMSO pH 6.8, 30 °C, quiescently. (c) Aggregation kinetics of 3 μM Aβ42 in the presence of 0 μM, 15 μM or 21 μM of YX-I-1. Aβ42 were performed in 20 mM sodium phosphate, 200 μM EDTA, pH 8.0, 1% (v/v) DMSO at 37 °C, quiescently. (d) Aggregation kinetics of 3 μM Aβ42 in the presence of 0 μM, 3 μM, or 6 μM of YX-A-1. Aβ42 were performed in 20 mM sodium phosphate, 200 μM EDTA, pH 8.0, 1% (v/v) DMSO at 37 °C, quiescently. (e) Aggregation kinetics of 10 μM Aβ42 in the presence of DMSO (black), 50 μM YX-I-1 (red), or 10 μM YX-A-1 (blue). Aβ42 aggregation kinetics were performed in 25 mM

sodium phosphate buffer containing 2% (v/v) DMSO pH 6.8, 30 °C, quiescently. Each experiment was performed in triplicate.

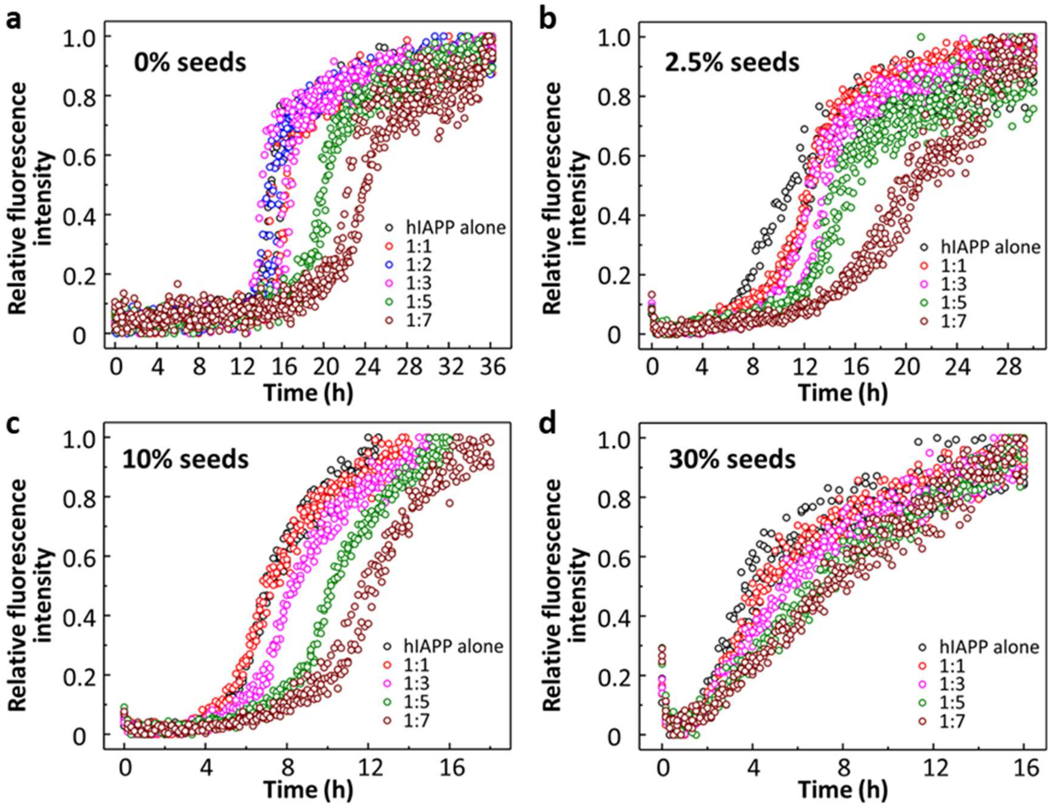

Supplementary Fig. 11. Effect of YX-I-1 on wt hIAPP aggregation in the absence or presence different concentrations of preformed fibril seeds. (a) Aggregation kinetics of 10 µM wt hIAPP without seeds in the presence of 0 µM, 10 µM, 20 µM, 30 µM, 50 µM or 70 µM of YX-I-1. Aggregation kinetics of 10 µM hIAPP with (b) 2.5% (v/v), (c) 10% (v/v) or (d) 30% (v/v) preformed fibril seeds in the presence of 0 µM, 10 µM, 30 µM, 50 µM or 70 µM of YX-I-1. Each experiment was performed in triplicate. For clarity, only a single kinetic curve taken from these data is shown in Fig. 5 of the main text. All reactions were performed in 25 mM sodium phosphate buffer containing 2% (v/v) DMSO pH 6.8, 30 °C, quiescently.

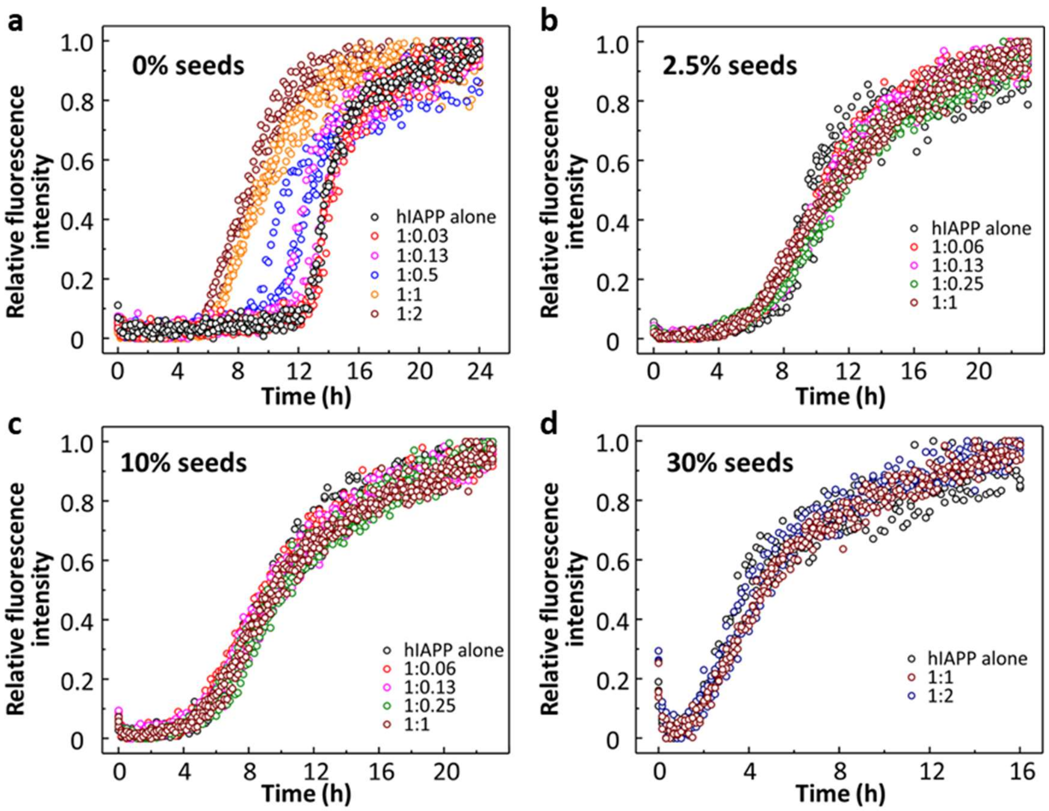

Supplementary Fig. 12. Effect of YX-A-1 on wt hIAPP aggregation in the absence or presence different concentrations of preformed fibril seeds. Aggregation kinetics of wt hIAPP (10 µM) (a) without seeds or in the presence of (b) 2.5% (v/v), (c) 10% (v/v), or (d) 30% (v/v) preformed fibril seeds, each in the presence of different concentrations of YX-A-1, as indicated (shown as wt hIAPP: YX-A-1 molar ratio in each plot). Each experiment was performed in triplicate. All reactions were performed in 25 mM sodium phosphate buffer containing 2% (v/v) DMSO pH 6.8, 30 °C, quiescently.

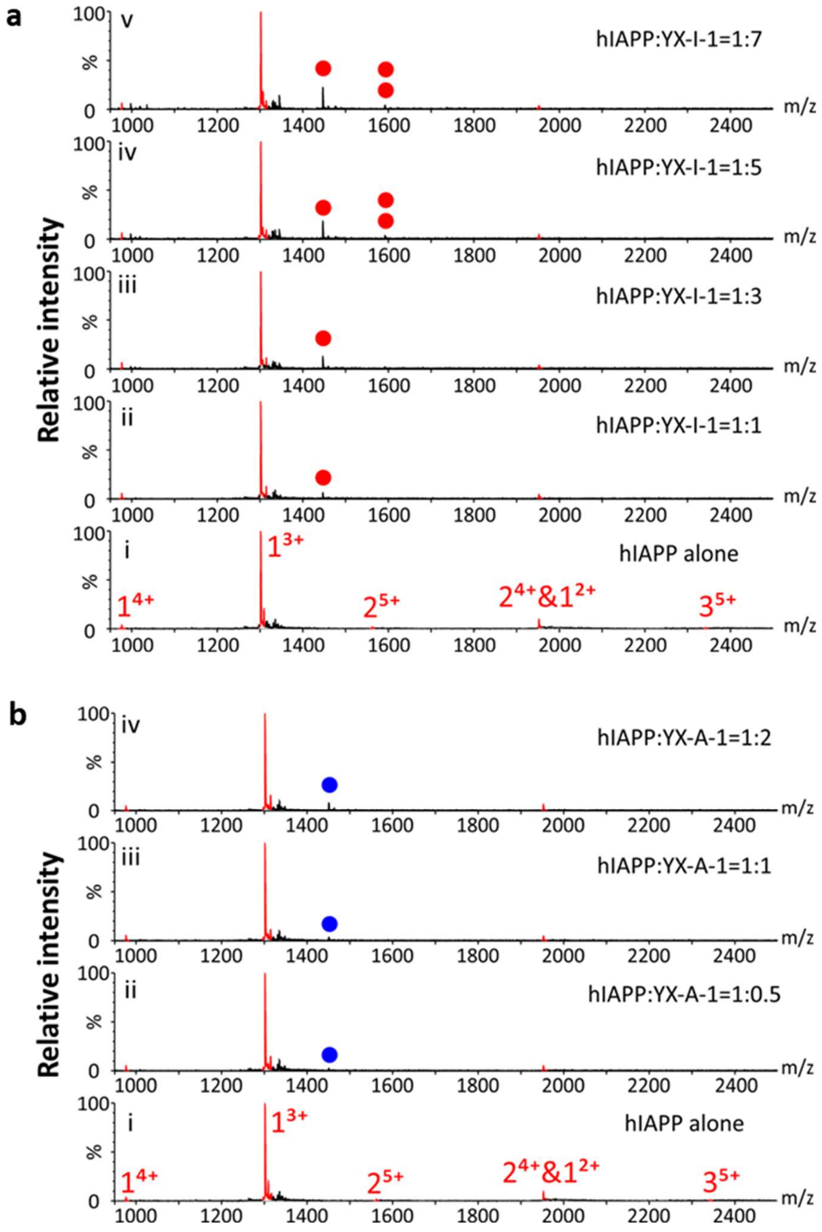

Supplementary Fig. 13. The interaction between wt hIAPP and YX-I-1 or YX-A-1 probed by nESI-MS. (a) Positive-ion native ESI mass spectra of 16 µM wt hIAPP in the presence of 2% (v/v) DMSO alone, or with different concentrations of YX-I-1 (molar concentration ratio of 1:1, 1:3, 1:5 or 1:7) show YX-I-1 binds to the 3+ charge state of monomeric wt hIAPP. (b) Positive-ion native ESI mass spectra of 16 µM wt hIAPP in the presence of 2% (v/v) DMSO, or different concentrations of YX-A-1 (molar concentration ratio of 1:0.5, 1:1 or 1:2) show YX-A-1 binds weakly (low ion intensity) to the 3+ charge state of monomeric wt hIAPP. Species of wt hIAPP are highlighted in red. Bound peaks are denoted with red circles (inhibitor) or blue circles (accelerator) and the number of circles represents the number of small molecules bound. Native mass spectrometry experiments were repeated more than three times (biological repeats).

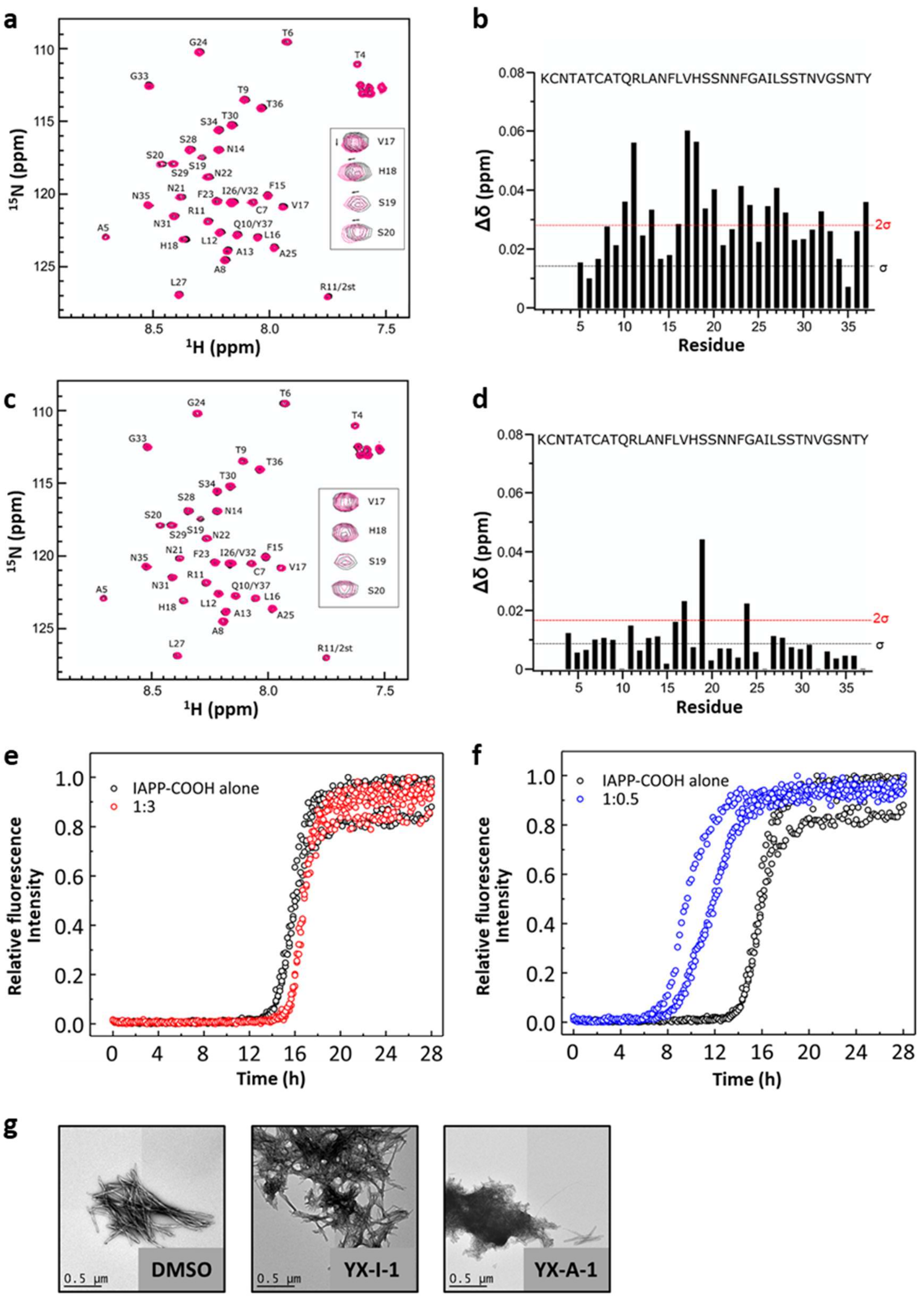

Supplementary Fig. 14. NMR analysis of the interaction between IAPP-COOH and YX-I-1/YX-A-1. (a) 2D ^{1}H-^{15}N SOFAST-HMQC spectra of 20 μM IAPP-COOH in the absence (black) or presence 100 μM YX-I-1 (purple). (b) ^{1}H-^{15}N-CSPs for the backbone amide resonances as a function of the amino acid sequence position for Supplementary Fig. 14a. (c) 2D ^{1}H-^{15}N SOFAST-HMQC spectra of 20 μM IAPP-COOH in the absence (black) or presence 20 μM YX-A-1 (purple). (d) ^{1}H-^{15}N-CSPs for the backbone amide resonances as a function of the amino acid sequence position for Supplementary Fig. 14c. The

black and red dash lines indicate one or two times of standard deviation. (e) Inhibition of IAPP-COOH aggregation by YX-I-1. Relative ThT fluorescence intensity of 10 μM IAPP-COOH in the absence (black) or presence of a 1:3 (red) molar ratio of IAPP-COOH to YX-I-1. (f) Acceleration of IAPP-COOH aggregation by YX-A-1. Relative ThT fluorescence intensity of 10 μM IAPP-COOH in the absence (black) or presence of a 1:0.5 (blue) molar ratio of IAPP-COOH to YX-A-1. (g) TEM images of IAPP-COOH incubated with DMSO, 30 μM YX-I-1 or 5 μM YX-A-1. The scale bar is 0.5 μm. The TEM images are consistent in all three experimental replicates. All reactions were performed in 25 mM sodium phosphate buffer (pH 6.8) containing 2% (v/v) DMSO at 30 °C, quiescently. NMR experiments were repeated twice. All the ThT kinetic experiments were performed in triplicate.

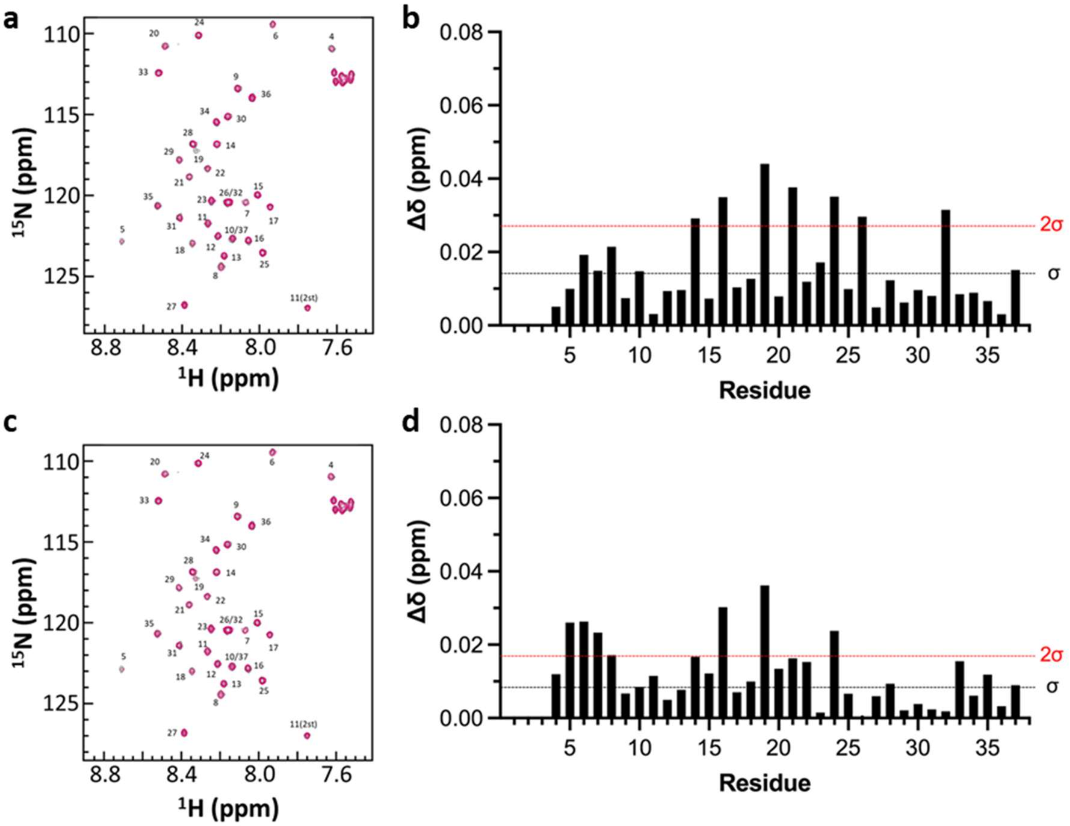

Supplementary Fig. 15. NMR analysis of the interaction between S20G and YX-I-1/YX-A-1. (a) 2D ^{1}H-^{15}N SOFAST-HMQC spectra of 20 μM S20G in the absence (black) or presence (purple) of 100 μM YX-I-1. (b) ^{1}H-^{15}N-CSPs for the backbone amide resonances as a function of the amino acid sequence position for Supplementary Fig. 15a. (c) 2D ^{1}H-^{15}N SOFAST-HMQC spectra of 20 μM S20G in the absence (black) or presence 20 μM YX-A-1 (purple). (d) ^{1}H-^{15}N-CSPs for the backbone amide resonances as a function of the amino acid sequence position for Supplementary Fig. 15c. The black and red dash lines indicate 1 or 2 standard deviation of the mean CSP for each experiment, respectively.

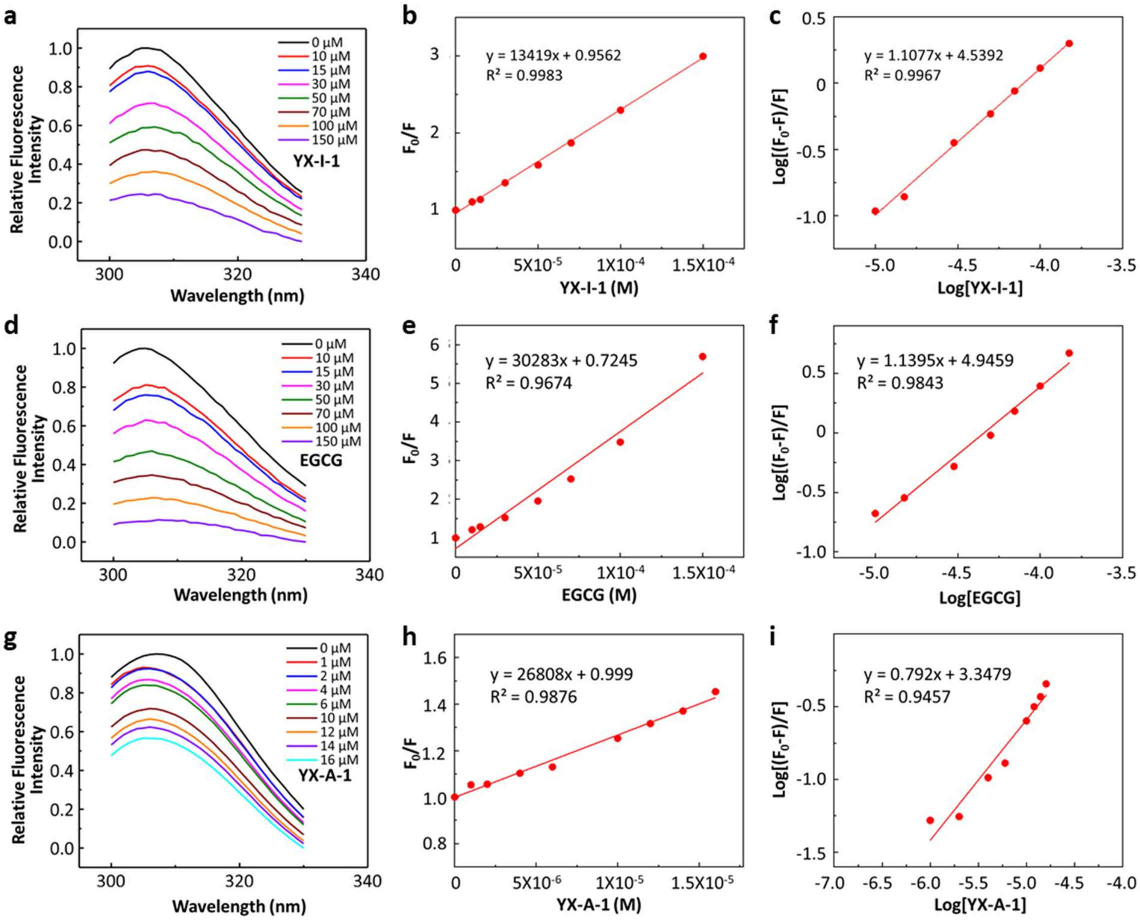

Supplementary Fig. 16. Fluorescence quenching analysis of the interaction between wt hIAPP and YX-I-1/YX-A-1/EGCG. The fluorescence emission spectra of 5 µM wt hIAPP in the absence or presence of various concentrations of YX-I-1 (a), EGCG (d), or YX-A-1 (g). The Stem-Volmer plot for the fluorescence quenching of wt hIAPP by YX-I-1 (b), EGCG (e), or YX-A-1 (h). The plot of log[(F_0−F)/F] against log[small molecule] for wt hIAPP. F_0 and F are the fluorescence intensities of wt hIAPP in the absence or presence of YX-I-1 (c), EGCG (f), or YX-A-1 (i).

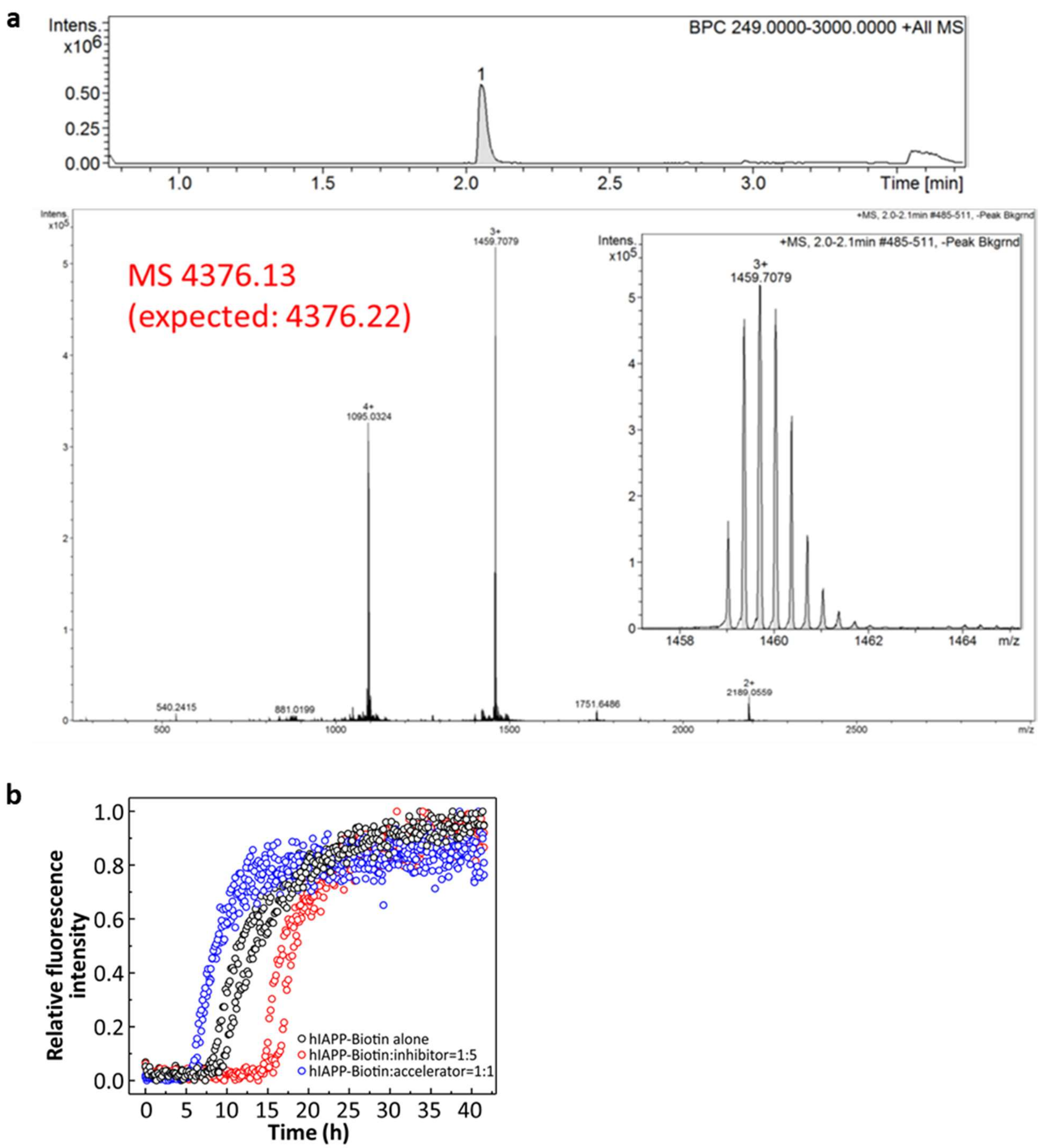

Supplementary Fig. 17. Characterisation of N-terminally biotinylated wt hIAPP. (a) LC-MS trace of biotinylated wt hIAPP. The expected and observed masses of the peptide containing a single biotin moiety (in Da) are shown. (b) Effect of inhibitor YX-I-1 and accelerator YX-A-1 on biotinylated wt hIAPP aggregation. Relative ThT fluorescence intensity of 10 µM biotinylated wt hIAPP in the absence (black) or presence of 50 µM YX-I-1 (red) or 10 µM YX-A-1 (blue). All reactions were performed in 25 mM sodium phosphate buffer (pH 6.8) containing 2% (v/v) DMSO at 30 °C, quiescently. All the ThT kinetic experiments were performed in duplicate.

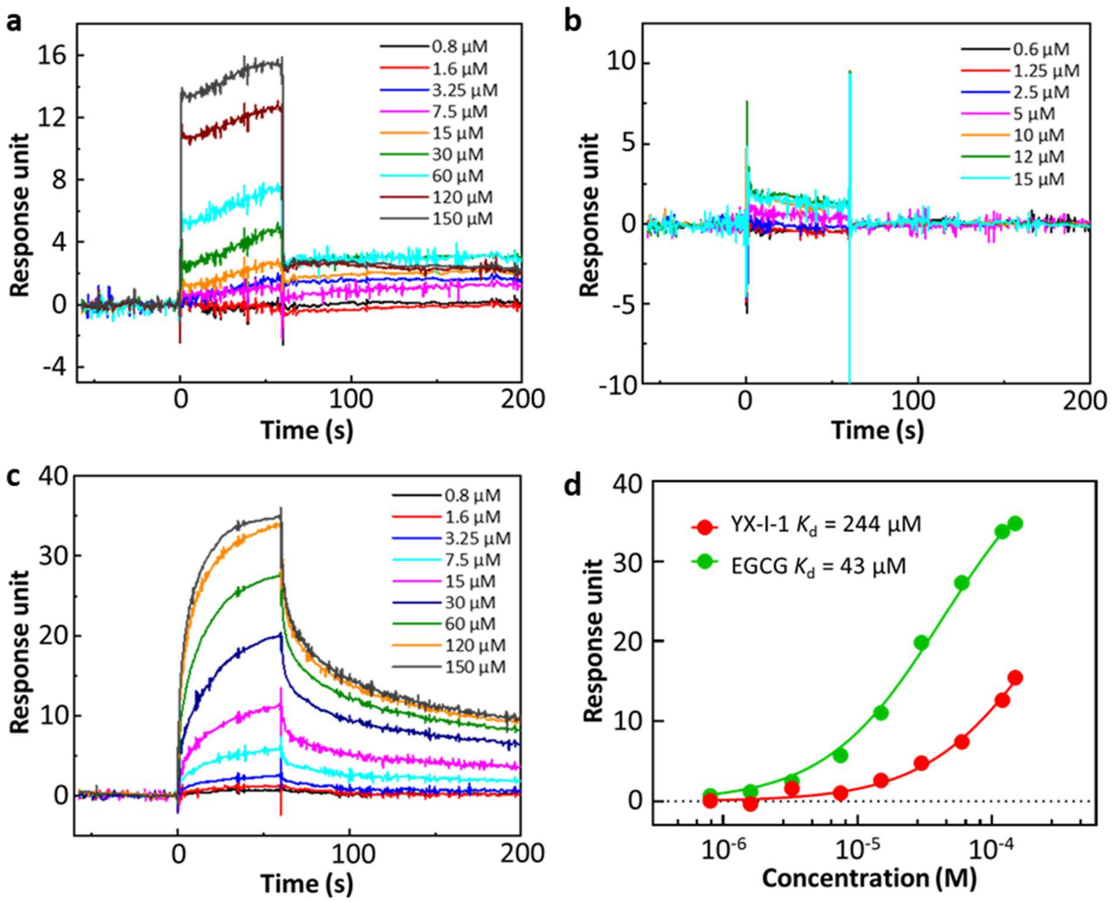

Supplementary Fig. 18. Sensorgrams and steady state plots for the binding of small molecules to wt hIAPP. Concentration-dependent binding sensorgram for (a) YX-I-1 and wt hIAPP, (b) YX-A-1 and wt hIAPP, and (c) EGCG and wt hIAPP. YX-I-1, YX-A-1 and EGCG were injected over wt hIAPP immobilised N-terminally to the surface for 60 s. (d) Steady state plots for the binding of YX-I-1 or EGCG to wt hIAPP. The steady state values were determined and plotted as a function of the concentration of small molecule added. A single binding site model was fitted to the data to estimate K_d. Note that the inability to saturate binding with YX-I-1 means that the K_d cannot be acutely defined using this technique, but confirms binding of the ligand to the peptide. SPR experiments were repeated twice.

Supplementary Table 1 Molecular properties of YX-I-1 and YX-A-1.

Compounds	MW[a]	HBD[b]	HBA[c]	clogP	Lipinski Violations
YX-I-1	435.5	2	8	3.07	0
YX-A-1	446.5	2	6	4.49	0
Lipinski's rule of five	⩽500	⩽5	⩽10	⩽5	

[a]Molecular weight; [b]Hydrogen bond donor; [c]Hydrogen bond acceptor

Supplementary Table 2 Stern–Volmer constant (K_{SV}), Quenching constant (k_q), binding constant (K_b), binding stoichiometry (n) and dissociation constant (K_d) for the interaction of YX-I-1, YX-A-1 and EGCG with wt hIAPP.

	K_{sv} (M^{-1})	k_q ($M^{-1}s^{-1}$)	R^2	K_b (M^{-1})	n	R^2	K_d (μM)
YX-I-1	1.34×10^4	1.34×10^{13}	0.9983	3.16×10^4	1.11	0.9967	32
EGCG	3.03×10^4	3.03×10^{13}	0.9674	7.94×10^4	1.14	0.9843	13
YX-A-1	2.68×10^4	2.68×10^{13}	0.9876	3.54×10^3	0.79	0.9457	280

Supplementary Note 1 | Characterisation of small molecule modulators

YX-I-1

^{1}H NMR (500 MHz, DMSO-d_6): δ 10.60 (s, 1H), 8.07 (d, *J* = 8.5 Hz, 1H), 7.83 (dd, *J* = 9.6, 8.5 Hz, 2H), 7.58 – 7.53 (m, 3H), 7.44 – 7.41 (m, 1H), 7.28 (d, *J* = 8.6 Hz, 2H), 5.68 (s, 2H), 4.37 (dd, *J* = 11.4, 2.1 Hz, 1H), 3.96 – 3.88 (m, 1H), 3.27 – 3.23 (m, 1H), 1.97 – 1.94 (m, 1H), 1.83 – 1.79 (m, 1H), 1.78 (s, 3H), 1.74 – 1.67 (m, 1H), 1.17 (q, *J* = 11.8 Hz,1H), 1.08 (q, *J* = 11.8 Hz, 1H), 0.92 (d, *J* = 6.8 Hz, 3H), 0.89 (d, *J* = 6.8 Hz, 3H).

^{13}C NMR (126 MHz, DMSO-d_6): δ 168.2, 164.3, 145.1, 138.4, 137.3, 133.9, 127.4, 126.0, 123.9, 119.1, 119.0, 111.0, 80.3, 76.5, 50.4, 45.7, 39.9, 34.0, 32.5, 22.7, 18.4, 18.3.

HRMS (m/z): [M+H]+ calcd. for $C_{24}H_{30}N_5O_3$, 436.2270; found, 436.2358;

YX-A-1

^{1}H NMR (500 MHz, DMSO-d_6): δ 10.48 (s, 1H), 9.00 (s, 1H), 8.48 – 8.46 (m, 2H), 7.92 – 7.84 (m, 1H), 7.80 (t, *J* = 5.7 Hz, 1H), 7.60 – 7.54 (m, 3H), 7.34 – 7.29 (m, 1H), 7.28 – 7.23 (m, 2H), 3.57 (q, *J* = 7.0 Hz, 2H), 3.13 (t, *J* = 7.0 Hz, 2H), 2.03 – 1.97 (m, 1H), 1.67 – 1.52 (m, 5H), 1.28 – 1.20 (m, 2H), 1.17 – 1.03 (m, 3H);

^{13}C NMR (126 MHz, DMSO-d_6): δ 175.2, 166.2, 164.6, 163.2, 156.1, 154.6 (d, *J* = 247.1 Hz), 136.7, 131.3, 128.8, 128.1, 127.6, 126.6 (d, *J* = 6.7 Hz), 125.5 (d, *J* = 6.7 Hz), 125.4, 124.4 (d, *J* = 3.4 Hz), 115.8 (d, *J* = 19.5 Hz), 43.9, 37.2, 34.9, 29.1, 25.4, 25.2;

HRMS (m/z): [M+H]+ calcd. for $C_{26}H_{28}FN_4O_2$, 447.2118; found, 447.2214;

YX-I-1

^{1}H NMR (500 MHz, DMSO-d_6)

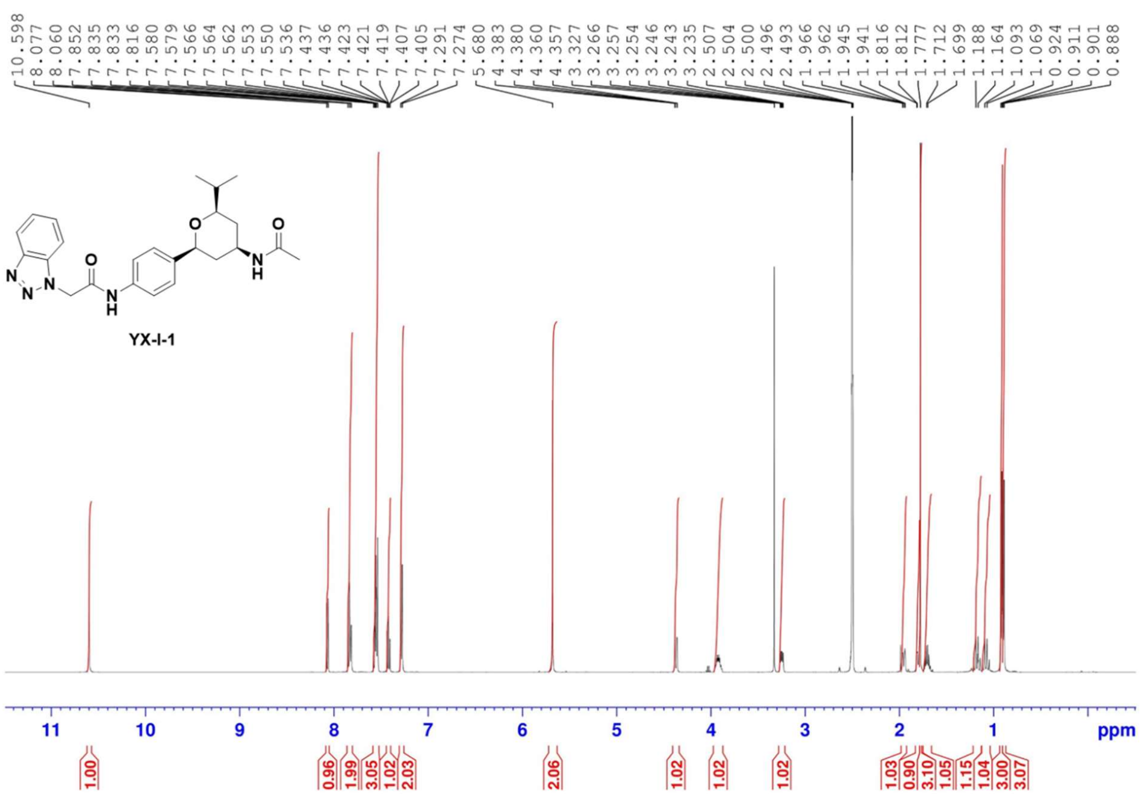

YX-I-1

^{13}C NMR (126 MHz, DMSO-d_6)

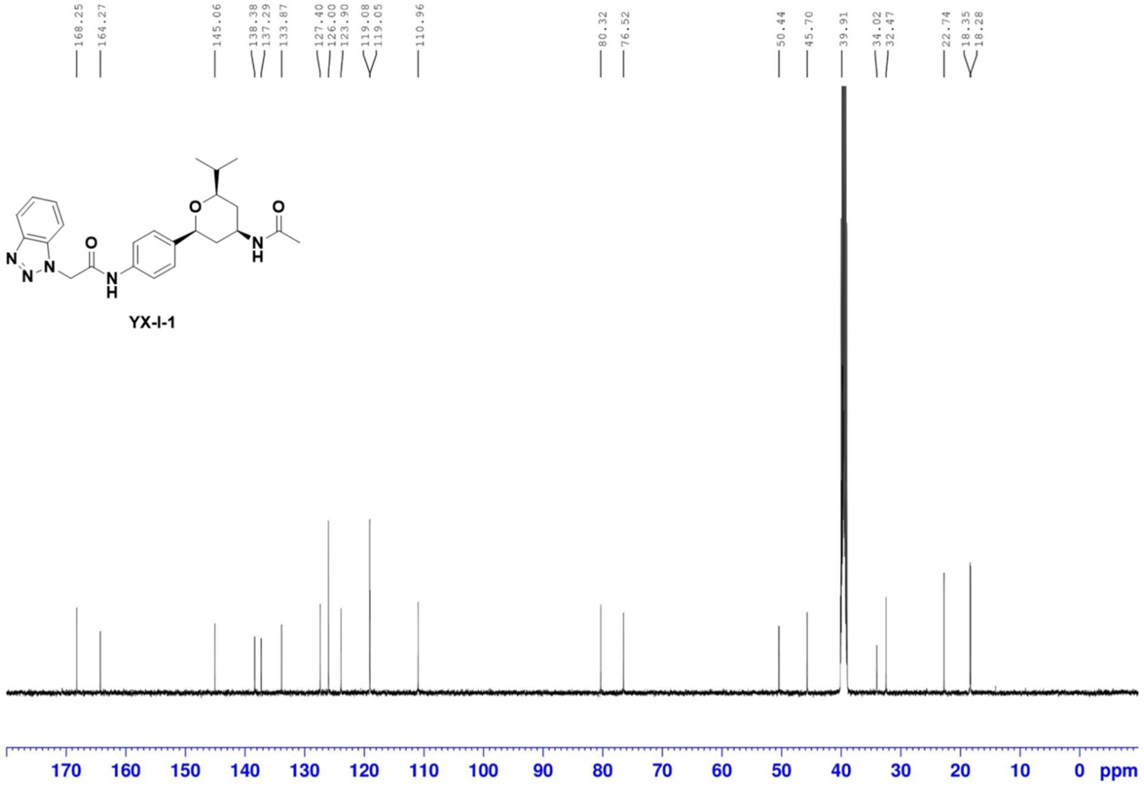

YX-A-1

^{1}H NMR (500 MHz, DMSO-d_6)

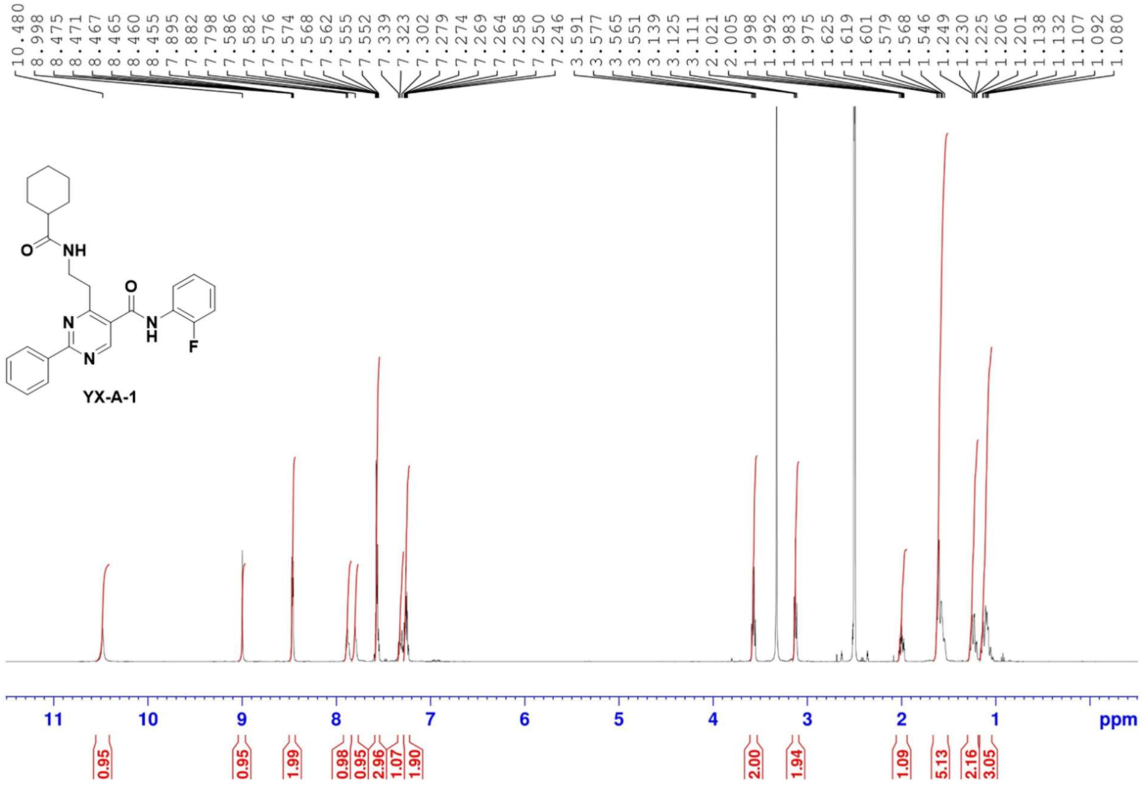

YX-A-1

^{13}C NMR (126 MHz, DMSO-d_6)

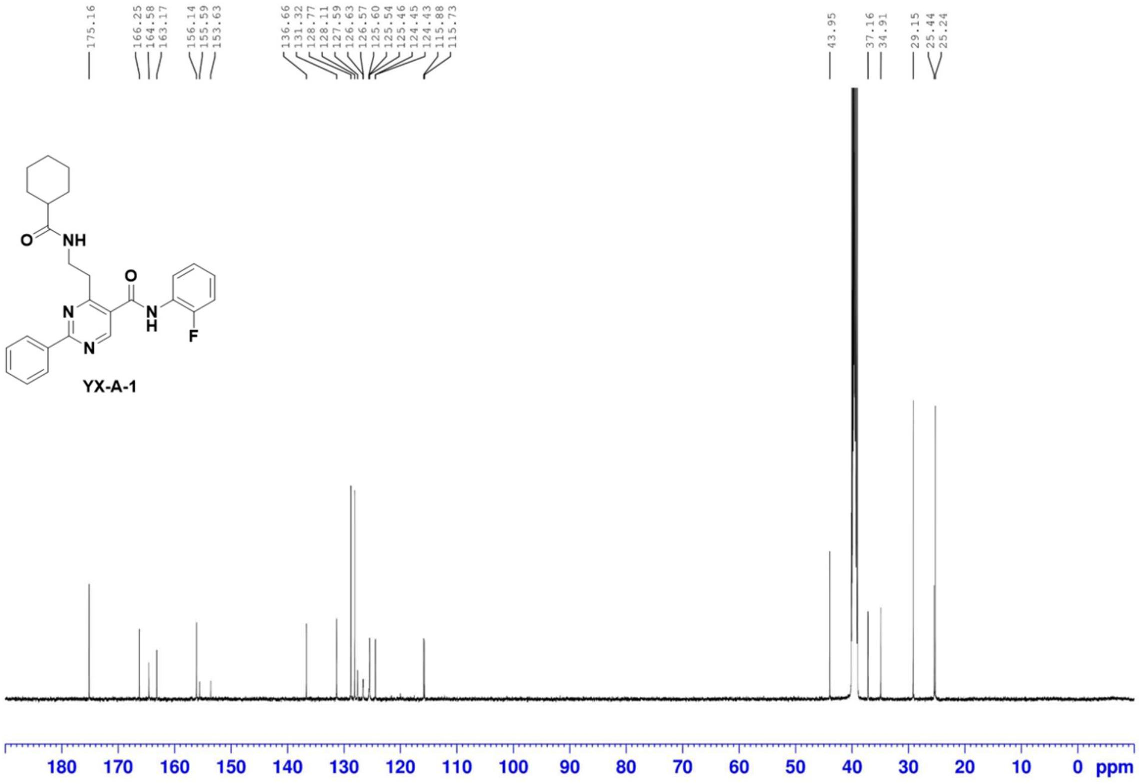

Supplement: Supplementary file 1 — Supplementary Information [file 41467_2022_28660_MOESM1_ESM.pdf]
