# Supplementary material for: Tuning the rate of aggregation of hIAPP into amyloid using small-molecule modulators of assembly
Source: Nat Commun. 2022 Feb 24;13:1040. doi: 10.1038/s41467-022-28660-7 (PMC8873464; doi:10.1038/s41467-022-28660-7)
Supplement: Supplementary file 2 — Reporting Summary [file 41467_2022_28660_MOESM2_ESM.pdf]

nature portfolio

Corresponding author(s): Prof Sheena E. Radford & Dr Richard Foster

Last updated by author(s): Jan 20, 2022

Reporting Summary

Nature Portfolio wishes to improve the reproducibility of the work that we publish. This form provides structure for consistency and transparency in reporting. For further information on Nature Portfolio policies, see our Editorial Policies and the Editorial Policy Checklist.

Statistics

For all statistical analyses, confirm that the following items are present in the figure legend, table legend, main text, or Methods section.

n/a	Confirmed	
☐	☒	The exact sample size (*n*) for each experimental group/condition, given as a discrete number and unit of measurement
☐	☒	A statement on whether measurements were taken from distinct samples or whether the same sample was measured repeatedly
☐	☒	The statistical test(s) used AND whether they are one- or two-sided<br>*Only common tests should be described solely by name; describe more complex techniques in the Methods section.*
☒	☐	A description of all covariates tested
☒	☐	A description of any assumptions or corrections, such as tests of normality and adjustment for multiple comparisons
☐	☒	A full description of the statistical parameters including central tendency (e.g. means) or other basic estimates (e.g. regression coefficient) AND variation (e.g. standard deviation) or associated estimates of uncertainty (e.g. confidence intervals)
☐	☒	For null hypothesis testing, the test statistic (e.g. *F*, *t*, *r*) with confidence intervals, effect sizes, degrees of freedom and *P* value noted<br>*Give P values as exact values whenever suitable.*
☒	☐	For Bayesian analysis, information on the choice of priors and Markov chain Monte Carlo settings
☒	☐	For hierarchical and complex designs, identification of the appropriate level for tests and full reporting of outcomes
☒	☐	Estimates of effect sizes (e.g. Cohen's *d*, Pearson's *r*), indicating how they were calculated

Our web collection on statistics for biologists contains articles on many of the points above.

Software and code

Policy information about availability of computer code

Data collection	ThT kinetics data were acquired using a plate reader (CLARIOstar or FLUOstar Omega, BMG Labtech).<br>TEM data were acquired using a JEM-1400 (JEOL Ltd) transmission electron microscope.<br>Native ESI-MS analysis was acquired using a Synapt G1 HDMS instrument (Waters Corp., Wilmslow, UK) equipped with a Triversa Nanomate (Advion Biosciences) automated nano-ESI interface.<br>NMR spectra were acquired on a Bruker Ascend AeonTM 950 MHz spectrometer equipped with a TCI-cryoprobe (3 mm).<br>Fluorescence quenching data were acquired on a Quantum Master Fluorimeter (Turret 400TM, Photon Technology) controlled by FelixGX software v4.3.<br>DLS data were acquired on a Wyatt miniDawn TREOS system, equipped with an additional quasi-elastic light scattering (QELS-synonym for DLS) detector.<br>SPR data were acquired on Biacore T200 (Cytiva).<br>AFM was acquired in Peak Force tapping mode using a Bruker Multimode 8 AFM with a Nanoscope V controller using Bruker PeakForce-HIRS-SSB probes.

Data analysis	The native MS data were analysed by using MassLynx V4.1.<br>All NMR spectra were processed and analysed using NMRPipeX and ccpNMR-Analysis (2.5.2) software.<br>The DLS data were analysed in Astra 6.1 software.<br>The quantitative chemical analysis was done by the online platform Amylofit.<br>Heights and lengths of fibrils were measured either automatically using MATLAB to trace along the fibril (http://github.com/George-R-Heath/Correlate-Filaments) or manually in ImageJ.<br>The SPR data were analysed by using BIA evaluation v2.0.<br>All the kinetic data were analysed by using Origin 2019b.<br>SPR data and kinetic data were analysed by using GraphPadd Prism 8.

For manuscripts utilizing custom algorithms or software that are central to the research but not yet described in published literature, software must be made available to editors and reviewers. We strongly encourage code deposition in a community repository (e.g. GitHub). See the Nature Portfolio guidelines for submitting code & software for further information.

Data

Policy information about availability of data

All manuscripts must include a data availability statement. This statement should provide the following information, where applicable:

- Accession codes, unique identifiers, or web links for publicly available datasets
- A description of any restrictions on data availability
- For clinical datasets or third party data, please ensure that the statement adheres to our policy

Source data are provided with this paper (all ThT kinetics, EM images, nESI-mass spectra, NMR, fluorescence quenching, UV absorbance, SPR data and AFM analysis) and are freely available at the University of Leeds Data Repository: https://doi.org/10.5518/1000.

Field-specific reporting

Please select the one below that is the best fit for your research. If you are not sure, read the appropriate sections before making your selection.

☒ Life sciences ☐ Behavioural & social sciences ☐ Ecological, evolutionary & environmental sciences

For a reference copy of the document with all sections, see nature.com/documents/nr-reporting-summary-flat.pdf

Life sciences study design

All studies must disclose on these points even when the disclosure is negative.

Sample size	No statistical methods were used to determine samples size. Experiments were repeated to ensure reproducibility.
Data exclusions	No data were excluded from the analysis.
Replication	Kinetic data were repeated at least twice with three replicates each time with different batches of the peptides. Native mass spec experiment were repeated at least three times. NMR experiments were repeated twice. The SPR experiment was repeated twice. Similar results were obtained for all the experiments.
Randomization	This is not relevant to our study, because no grouping was needed.
Blinding	Investigators were not blinded to group allocation, as no grouping was needed for this study.

Reporting for specific materials, systems and methods

We require information from authors about some types of materials, experimental systems and methods used in many studies. Here, indicate whether each material, system or method listed is relevant to your study. If you are not sure if a list item applies to your research, read the appropriate section before selecting a response.

Materials & experimental systems

n/a	Involved in the study	
☒	☐	Antibodies
☒	☐	Eukaryotic cell lines
☒	☐	Palaeontology and archaeology
☒	☐	Animals and other organisms
☒	☐	Human research participants
☒	☐	Clinical data
☒	☐	Dual use research of concern

Methods

n/a	Involved in the study	
☒	☐	ChIP-seq
☒	☐	Flow cytometry
☒	☐	MRI-based neuroimaging
